# Condensed-matter equation of states covering a wide region of pressure studied experimentally

**DOI:** 10.1038/srep39212

**Published:** 2016-12-15

**Authors:** Elijah E. Gordon, Jürgen Köhler, Myung-Hwan Whangbo

**Affiliations:** 1 Department of Chemistry, North Carolina State University, Raleigh, NC 27695-8204, USA; 2 Max-Planck-Institut für Festkörperforschung, D-70569 Stuttgart, Germany

## Abstract

The relationships among the pressure *P*, volume *V*, and temperature *T* of solid-state materials are described by their equations of state (EOSs), which are often derived from the consideration of the finite-strain energy or the interatomic potential. These EOSs consist of typically three parameters to determine from experimental *P-V-T* data by fitting analyses. In the empirical approach to EOSs, one either refines such fitting parameters or improves the mathematical functions to better simulate the experimental data. Despite over seven decades of studies on EOSs, none has been found to be accurate for all types of solids over the whole temperature and pressure ranges studied experimentally. Here we show that the simple empirical EOS, *P = α^1^*(*PV*) + *α^2^*(*PV*)*^2^* + *α^3^*(*PV*)*^3^*, in which the pressure *P* is indirectly related to the volume *V* through a cubic polynomial of the energy term *PV* with three fitting parameters *α^1^*–*α^3^*, provides accurate descriptions for the *P*-vs-*V* data of condensed matter in a wide region of pressure studied experimentally even in the presence of phase transitions.

One of the most important issues in condensed matter sciences, particularly, in geology and geophysics, is to accurately predict the structural and physical properties of solids under high pressure and temperature^1^,^2^,^3^,^4^,^5^,^6^,^7^. In general, a solid-state material under high pressure and temperature can exhibit properties quite different from those found at ambient conditions. At a given temperature *T*, a solid under external pressure *P* decreases its volume *V* with increasing *P*, but *V* changes a lot more slowly than does *P*. The pressure-induced volume decrease may require a change in the structure type (i.e., the pattern of the relative atom arrangements in a repeat unit cell) thereby causing a structural phase transition and an associated physical property change. For example, when *P* is increased at room temperature, elemental chalcogen Te^8^,^9^,^10^,^11^,^12^,^13^,^14^, Se^15^,^16^,^17^,^18^,^19^,^20^, or S^15^,^21^,^22^,^23^,^24^,^25^ undergoes a number of structural phase transitions while its electrical property changes from insulating at ambient pressure to metallic and superconducting at high pressure^26^,^27^. Hydrogen sulfide H_2_S is a diamagnetic molecular species at ambient conditions, but is converted, under the pressure of over ∼110 GPa, to a condensed phase that becomes superconducting at ∼200 K^25^,^28^, the highest among all superconductors known so far. An isothermal EOS relates *P* and *V* at a certain temperature *T*. Over the past 70 years the *P*-vs-*V* data have been studied for a variety of solids in various pressure ranges (e.g., see Table 1), and their EOSs have been examined. So far, however, no isothermal EOS is applicable to all types of solids and is accurate over the whole range of pressure studied especially when a solid undergoes several structural phase transitions in the pressure region studied.

With increasing pressure *P*, the volume *V* of a solid under pressure changes very slowly compared with the pressure change. The shortcomings of the known EOSs originate essentially from the attempts to relate the fast changing variable *P* to a very slowly changing variable *V*. These problems can be circumvented if the pressure change is related to a pressure-induced energy change that is associated with the volume *V* and also varies nearly at the same rate as does *P*. The energy term *PV* satisfies these two requirements because, while increasing *P*, the volume *V* of a solid under pressure *P* decreases very slowly so that the term *PV* changes nearly as fast as *P* in the entire range of *P*. Furthermore, at a given *P*, the term *PV* is determined by the value of *V*, not by how the atoms are arranged within the volume so that the term *PV* cannot be overly sensitive to phase transitions. With increasing *P*, the term *PV* should increase slightly more slowly than does *P* because *V* decreases slightly under pressure. Therefore, it should be possible to accurately describe the *P*-vs-*V* data of any solid over the entire pressure range studied experimentally by the simple EOS,


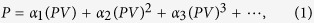


which expands *P* as a polynomial of *PV*, where the constants α_i_ (i = 1, 2, 3, etc.) are the fitting parameters. For those familiar with the traditional EOSs, use of Eq. 1 is quite unconventional because *P* is expanded in terms of the variable *PV* that contains itself. However, our goal is to find an accurate, though indirect, relationship between *P* and *V* valid for the entire pressure region studied experimentally by way of determining an accurate relationship between *P* and *PV*. From the resulting *P*-vs-*V* relationship, one can derive an accurate description of other thermodynamic quantity such as bulk modulus, as we demonstrated in our work. When this expression is truncated to *P* = α_1_(*PV*) + α_2_(*PV*)^2^, *P* = α_1_(*PV*) + α_2_(*PV*)^2^ + α_3_(*PV*)^3^, and *P* = α_1_(*PV*) + α_2_(*PV*)^2^ + α_3_(*PV*)^3^ + α_4_(*PV*)^4^, we obtain the quadratic, cubic and quartic approximations for Eq. 1. Here we establish that the cubic approximation with three fitting parameters α_1_–α_3_ is accurate enough in describing the *P*-vs-*V* data for condensed matter in a wide region of pressure experimentally probed even in the presence of several structural phase transitions.

## Results

### Formulation of the EOS

In testing whether an isothermal EOS is accurate over the entire range of pressure studied experimentally, the ideal systems to analyze would be elemental chalcogens Te, Se and S because they have been studied at room temperature over wide pressure ranges (i.e., 0–330 GPa for Te, 0–150 GPa for Se, and 0–213 GPa for S) (see Table 1), because each chalcogen undergoes a number of phase transitions with increasing *P*, and because their room-temperature atomic structures are known under widely different pressures. For each chalcogen, we begin our analysis by first determining the relative energies of its known atomic structures at various *P* on the basis of density functional theory (DFT) calculations, the details of which are described in Methods. We summarize the space groups of the known atomic structures at various pressures *P* (mostly around the room temperature), the volumes *V* per atom, the energies *PV* per atom, and the calculated electronic energies *E* per atom in Section 1(a)–(c) of the supporting information (SI). The calculated electronic structures are also presented in terms of density of states (DOS) plots in Section 1(d)–(f) of the SI. As anticipated, with increasing pressure, the DOS plot for each chalcogen is shifted toward the higher energy while the band gap present at low pressure disappears at high pressure.

**Figure 1:**
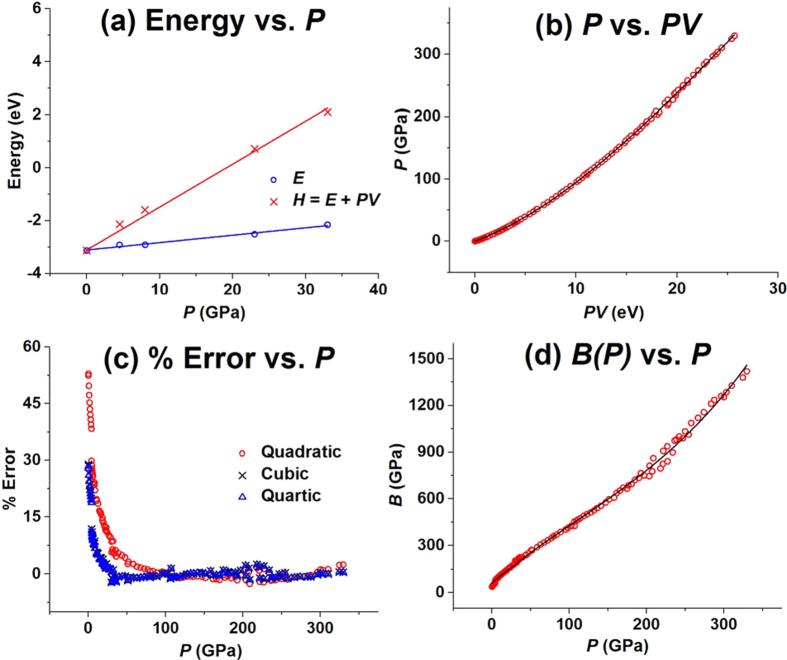
(**a**) The *E*-vs- *P* and *H*-vs-*P* plots calculated for Te, where *E, H* and *PV* are in eV units. The fitting coefficients *a*_1_, *a*_0_ and *b*_1_ for the linear plots *E* = *a*_1_*P* *+* *a*_0_ and *H* = *b*_1_*P* + *a*_0_ are respectively −3.0238, 0.0246 and 0.1589. (**b**) The *P*-vs-*PV* plot constructed from the experimental *P*-vs-*V* data for Te, where the solid line is the fitting curve obtained by using the cubic approximation for the EOS, Eq. 1. (**c**) The pressure-dependence of the % error, 100 × (*P*_calc_–*P*_expt_)/*P*_expt_, of the *P*-vs-*PV* plot obtained for Te by using the cubic approximation of the EOS, Eq. 1. (**d**) The pressure dependence of the bulk modulus *B*(*P*) calculated for Te.

The calculated energy *E* for Te is plotted as a function of *P* in Fig. 1a (those for Se and S in Section 2(a,b) of the SI), which shows a reasonable linear relationship, *E*≈*a*_*1*_*P* *+* *a*_*0*_, with slope *a*_1_ and intercept *a*_0_. Fig. 1a also plots the enthalpy *H* = *E* + *PV* per atom as a function of *P* using *a*_0_ as the intercept. This plot also exhibits a reasonable linear relationship, *H*≈*b*_*1*_*P* *+* *a*_*0*_, with the slope *b*_1_ > > *a*_1_ for all chalcogens. At a given temperature, therefore, the energy term *PV* = *H*–*E* varies almost linearly with *P*, i.e., *P*≈*PV/*(*b*_*1*_–*a*_*1*_). Unlike the case of gaseous substances for which the *PV* term is a constant independent of *P* at a given *T*, the *PV* term for each condensed-phase chalcogen increases almost linearly with *P* because, compared with the rate of change in *P*, that in *V* is very small. Nevertheless, the *H*-vs-*P* plot for each chalcogen is slightly concave down with respect to the base line, *b*_*1*_*P*. As already pointed out, this reflects that, with increasing *P*, the rate of change in *P* is slightly greater than that in *PV* due to a pressure-induced decrease in *V*. The latter allows one to expand *P* as a power series of *PV* as expressed in Eq. 1. Indeed, the *P*-vs-*V* data points used for constructing the *H*-vs-*P* plots are very well described, for example, by the quadratic approximation of Eq. 1 (see Section 2(c) of the SI). As will be discussed below, the nonlinear terms of Eq. 1, e.g., α_2_(*PV*)^2^ and α_3_(*PV*)^3^, are related to how the volume *V* of a solid decreases under pressure *P*.

To test the applicability of the isothermal EOS, Eq. 1, in a wide region of pressure studied experimentally, we first analyze the experimental *P*-vs-*V* data available in the literature for each chalcogen. The *P*-vs-*PV* plot for Te, presented in Fig. 1b, reveals that the experimental points in the 0–330 GPa region^8^,^9^,^10^,^11^,^12^,^13^,^14^ are very well described by the cubic approximation. The fitting curves from the quadratic and quartic approximations are not shown because they are practically impossible to distinguish, with naked eye alone, from that of the cubic approximation. The same conclusion is reached for Se^15^,^16^,^17^,^18^,^19^,^20^ and S^15^,^21^,^22^,^23^,^24^,^25^ (see Section 2(a,b) of the SI for Se and S, respectively). The fitting coefficients α_1_–α_3_ obtained for Te, Se and S resulting from the cubic approximation are summarized in Table 2, and those from the quadratic, cubic and quartic approximations are compared in Section 2(d) of the SI. The coefficients α_1_ and α_2_ are always positive, and α_1_ > > α_2_ > > |α_3_| with α_2_/α_1_ ≈ 10^−3^ and |α_3_|/|α_2_| ≈ 10^−4^.

### Error analysis

To assess the accuracies of the quadratic, cubic and quartic approximations for Eq. 1, we analyze the pressure-dependence of the absolute errors, Δ*P* = *P*_calc_–*P*_expt_, as well as that of the % errors, 100 × Δ*P*/*P*_expt_, where *P*_expt_ is the pressure observed experimentally, and *P*_calc_ the one calculated from the fitting equations. The pressure-dependence of the % errors for Te is shown in Fig. 1c, and those for Se and S in Section 2(a,b) of the SI. The maximum % error is smaller than 5.5% in the 10.9–330 GPa region for the cubic and quartic approximations, but smaller than 5.4 % in the 38–330 GPa region for the quadratic approximation. The % errors are large in the low *P* region, but it should be pointed out that the associated absolute errors are rather small (for example, for *P*_expt_ = 0.98 GPa, the corresponding *P*_calc_ values are 1.44, 1.22 and 1.22 GPa from the quadratic, cubic and quartic approximations, respectively) (see Section 2(e) of the SI). In general, the cubic and quartic approximations are similar in accuracy, and are more accurate than the quadratic approximation especially in the low *P* region. A similar conclusion is reached from the % error and absolute error plots calculated for Se in the 0–140 GPa range and for S in the 0–213 GPa range (see Section 2(a,b) of the SI).

### Applicability to other condensed matter

**Figure 2:**
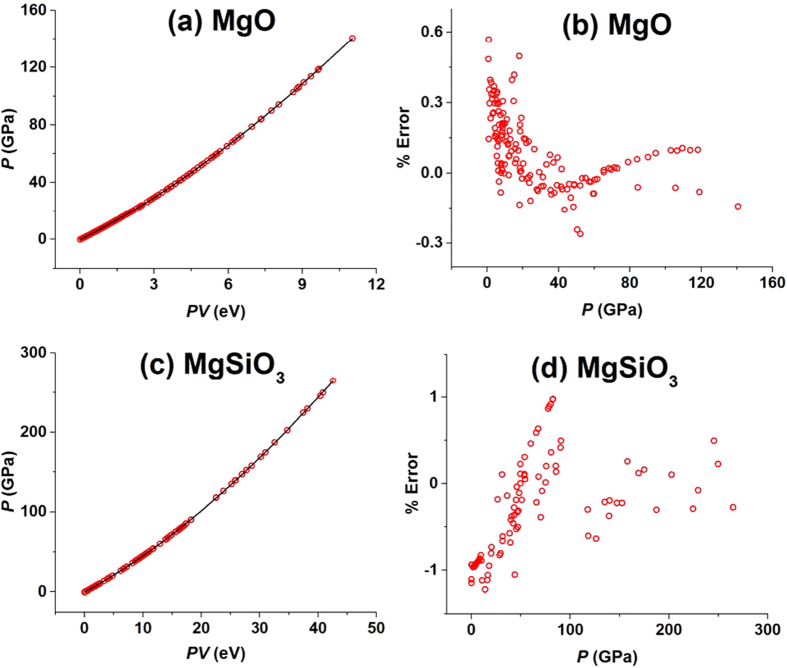
(**a,c**) The *P*-vs-*PV* plots constructed from the experimental *P*-vs-*V* data for MgO and MgSiO_3_, respectively, where the solid lines are the fitting curves obtained by using the cubic approximation of the EOS, Eq. 1. (**b,d**) The plots of the % errors vs. pressure obtained for MgO and MgSiO_3_, respectively, by using the cubic approximation of the EOS, Eq. 1.

The above analyses of the experimental *P*-vs-*V* data for elemental chalcogens suggest strongly that the cubic approximation of the isothermal EOS, Eq. 1, can accurately describe the experimental *P*-vs-*V* data for various solids in the whole pressure range studied experimentally. To establish this point, we examine the experimental *P*-vs-*V* data for various solid-state condensed matter listed in Table 1, which include the elemental Sn, the transition-metals Au and Cu, the alkali halides LiF, NaF, NaCl and CsCl, ice VII, the oxides MgO and MgSiO_3_, the noble gases Ar, Kr and Xe, as well as molecular hydrogen H_2_D_2_. As representative examples of these analyses, we discuss the oxides MgO^29^,^30^,^31^,^32^,^33^,^34^,^35^ and MgSiO_3_^36^,^37^,^38^,^39^,^40^,^41^. The isothermal *P*-vs-*V* relationships of these oxides have been extensively studied because they are the end members of (Mg,Fe)O^42^,^43^ and (Mg,Fe)SiO_3_ perovskite^42^,^44^, which are the important components of the Earth’s lower mantle. The *P*-vs-*V* relationships for MgO^29^,^30^,^31^,^32^,^33^,^34^,^35^ were examined at room temperature in the 0–140 GPa range, and those for MgSiO_3_^36^,^37^,^38^,^39^,^40^,^41^ at room temperature in the 0–300 GPa range. The *P*-vs-*PV* plots and the % error vs. *P* plots for MgO and MgSiO_3_ are presented in Fig. 2. The maximum % error is smaller than ∼0.6% for MgO, and smaller than ∼1% for MgSiO_3_, in the entire pressure ranges studied experimentally. For the remainder of the solids listed in Table 1, our results are summarized in Section 3(a)–(k) of the SI. The fitting coefficients α_1_–α_3_ obtained for the solid-state condensed matter of Table 1 from the cubic approximation are listed in Table 2 together with the maximum % errors. It is clear that the cubic approximation of the isothermal EOS, Eq. 1, provides an accurate description in the entire pressure regions examined experimentally. (Hereafter, the cubic approximation of Eq. 1 will be used without further mentioning).

The isothermal EOS, Eq. 1, is also applicable to non-solid-state condensed matter. As examples, we analyze the experimental *P*-vs-*V* data for the polymer, poly(ε-caprolactone) (PCL)^45^, determined at 100.6 °C in the 0–0.2 GPa region as well as those for liquid H_2_O^46^ determined at 15 °C, 25 °C and 35 °C in the 0–0.1 GPa region. Our results summarized in Section 3(m)–(p) of the SI show that the maximum % error for the polymer is smaller than 0.3%, and that for liquid water is practically zero, in the entire pressure region studied. Clearly, Eq. 1, provides an accurate description of the *P*-vs-*V* relationship for these materials. It should be pointed out that the ideal gas law is a special case of Eq. 1, when the PV term is a constant independent of *P*.

## Discussion

### Bulk modulus

Now that Eq. 1 provides an isothermal EOS accurate for the entire pressure range studied for a given system, we search for a simple expression for the corresponding bulk modulus *B* valid for the entire pressure region. In the cubic approximation, Eq. 1 is a quadratic equation of *P*, from which *P* is written in terms of *V* as


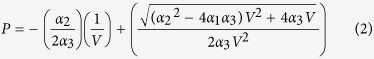


Thus, the bulk modulus *B* is expressed as


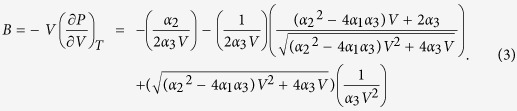


This equation expresses the bulk modulus as a function of volume, namely, *B*(*V*). For each value of *V*, however, there is a unique value of *P* associated with it, so that the *B*(*V*) vs. *V* relationship can be easily converted to the corresponding *B*(*P*) vs. *P* relationship. For convenient use of this relationship, we fit the *B*(*P*)-vs.-*P* relationship by the polynomial,


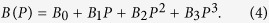


The *B*(*P*)-vs-*P* plot thus-obtained for Te is presented in Fig. 1d. For other solids listed in Table 1, the *B*(*P*)-vs-*P* plots are presented in Section 3(a)–(r) of the SI. The coefficients *B*_0_, *B*_1_, *B*_2_ and *B*_3_ determined for each condensed matter are summarized in Table 3, which also lists the calculated bulk modulus at *P* = 0, referred to as *B*_0,calc_, for each system. The *B*_0_ deviates from the *B*_0,calc_ because the polynomial fitting (Eq. 4) poorly describe the low-pressure region. Nevertheless, the *B*_0_ and *B*_0,calc_ values are quite similar for all systems except for Te and Se.

For every system, our analysis leads to only one *B*_0_ value because the entire pressure region studied is represented by the single fitting curve, Eq. 4. In the traditional study for a system undergoing several phase transitions, each phase covering a certain pressure region (say, *P*_1_ to *P*_2_) is described by the EOS covering only the pressure region *P*_1_–*P*_2_. The resulting EOS for each different phase generates the bulk modulus, which we will refer to as *B*_0,expt_, and the virtual volume *V*_0_. It is the *B*_0,expt_ obtained for the “first” phase (i.e., the phase stable in the lowest pressure region for which *P*_1_ = 0) that should be compared with the *B*_0_ or the *B*_0,calc_ value obtained from our EOS. For systems with several phases, Table 3 lists only the *B*_0,expt_ values of their first phases. Clearly, these *B*_0,expt_ values are well described by the *B*_0_ and/or *B*_0,calc_ values determined from our EOS analyses. The *B*_0,expt_ values found for the various phases of Te, Se and S can be accounted for in terms of our EOS analyses as presented in Section 4 of the SI.

### Qualitative meaning of the EOS

To gain insight into the meaning of the EOS, Eq. 1, we rewrite it in a slightly different form. In general, α_1_(*PV*) > > α_2_(*PV*)^2^ > > α_3_(*PV*)^3^ so that *P*≈α_1_(*PV*). By using this approximation, Eq. 1 is rewritten as


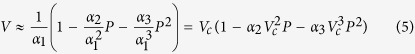


where the volume *V*_c_ is defined as *V*_c_ ≡ 1/α_1_, which is very close to the volume at zero-pressure, *V*_0_. Eq. 5 reveals that the decrease in the volume of a condensed matter under pressure is a polynomial function of *P*. Since α_2_(*PV*_c_)^2^ > > |α_3_|(*PV*_c_)^3^, the term α_2_*V*_c_^2^*P* dominates over α_3_*V*_c_^3^*P*^2^. Namely, the volume decreases with increasing *P*. The α_3_*V*_c_^3^*P*^2^ term compensates the overcorrection (when α_3_ < 0), or the under-correction (when α_3_ > 0), given by the α_2_*V*_c_^2^*P* term. In essence, the EOS, Eq. 1, reveals that the volume of a solid under pressure can be described as a polynomial function of pressure.

### Summary

We presented the empirical equation of state that accurately describes the pressure-versus-volume data for various types of condensed matter in a wide region of pressure studied experimentally. This is also true for systems undergoing several phase transitions in the pressure region studied. This equation of states results from the fact that the pressure change of a condensed matter is accurately described by a cubic polynomial of the pressure times the volume.

## Methods

Our non-spin-polarized DFT calculations employed the frozen-core projector augmented wave method^47^,^48^ encoded in the Vienna ab initio simulation package^49^, and the generalized-gradient approximation of Perdew, Burke and Ernzerhof 50 for the exchange-correlation functional. To ensure the accuracies of the calculations, a high plane-wave cut-off energy of 1000 eV was used, and the Brillouin zone associated with each repeat unit cell was sampled by a large number of k-points. For example, the 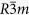 structure of S at 206 GPa was calculated by using a set of 24 × 24 × 24 k-points. Our calculations for Te, Se and S employed their reported crystal structures under various pressures, except for the 160 and 173 GPa structures of S as described in Section 1(c). The threshold for the self-consistent-field energy convergence was set at 10^−8^ eV for all structures of Se, Te and S. For the optimization of the structures of S at 160 and 173 GPa, the threshold for the force convergence at each atom was set at 0.005 eV/Å.

## Additional Information

**How to cite this article**: Gordon, E. E. *et al*. Condensed-matter equation of states covering a wide region of pressure studied experimentally. *Sci. Rep.* **6**, 39212; doi: 10.1038/srep39212 (2016).

**Publisher's note:** Springer Nature remains neutral with regard to jurisdictional claims in published maps and institutional affiliations.

## Supplementary Material

Supplementary Information

## Figures and Tables

**Table 1:** The pressure ranges (in GPa) and temperature (K) employed to examine the isothermal *P*-vs-*V* relationships for various condensed matter†,‡.

System	Pressure range (GPa)	Temperature (K)
Te	0^11^, 0–4^10^, 4.5^12^, 8^13^, 4–36^9^, 33^14^, 30–330^8^	298
Se	0^17^, 0–10^15^, 4.6^18^, 23^19^, 28,^19^, 87.9^20^, 140^19^, 5–150^16^	293–298
S	0^22^, 0–30^15^, 35–87^15^, 89.4^23^, 145^21^, 160^24^, 173^25^, 206.5^21^, 88–213^21^	293–298
Sn	0–120^a–c^	298
Au	4–70^d^	298
Cu	7–95^e^	293
LiF	0–4^h^, 1–9^f^, 0–30^g^	298
NaF	1–9^f^, 0–38^g^	298
NaCl	0–4^i^	298
CsCl	0–5^k^, 1–9^f^, 0–45^j^	298, 293
Ice VII	3–19^10^, 4–128^m^	300
MgO	0–8^33^, 0–11^30^, 0–20^32^, 0–24^31^, 0–52^29^, 4–120^35^, 10–140^34^	298
MgSiO_3_	0–10^40^, 0–20^36^, 0–55^37^, 36–83^38^, 29–91^39^, 100–300^41^	298
Ar	0–2^6^^,n^	40
Kr	0–2^6^^,n^	60
Xe	0–2^6^^,n^	60
H_2_	0–2.6^47^	4.2
D_2_	0–2.6^47^	4.2
PCL	0–0.2^45^	373.6
Liquid H_2_O	0–0.1^46^	288
Liquid H_2_O	0–0.1^46^	298
Liquid H_2_O	0–0.1^46^	308

^†^In our analysis for Sn, the α-Sn phase was excluded because it exists below 286 K, but all other phases of Sn that exist at room temperature are included.

^‡^For the references a–p, see Section 3 of the SI.

**Table 2:** The coefficients α_1_–α_3_ of the isothermal EOS, *P* = α_1_(*PV*) + α_2_(*PV*)^2^ + α_3_(*PV*)^3^, obtained for various condensed matter.

	10^2^ × α_1_	10^5^ × α_2_	10^9^ × α_3_	*P* range	Max. % error^b^
(a) Solid-state condensed matter (*P* in GPa, and *V* in Å^3^)					
Te	3.785	1.501	−1.130	0–330	5.5 (*P* > 10.9) 29 (*P* < 10.9)
Se	4.782	2.421	+1.325	0–150	3.6 (*P* > 23) 44 (*P* < 23)
S^a^	3.587	7.987	−16.20	0–213	4.6 (*P* > 36) 13 (*P* < 36)
Sn	3.978	1.897	−2.039	0–120	2.2 (*P* > 10.3) 8.0 (*P* < 10.3)
Au	5.839	2.079	−4.474	4–70	1.9
Cu	8.543	4.582	−10.02	7–95	1.0 (*P* > 56) 6.5 (*P* < 56)
LiF	6.064	6.646	−59.75	0–30	0.98
NaF	4.068	2.922	−13.88	0–38	1.5
NaCl	2.232	2.006	−11.35	0–4	0.1
CsCl	1.518	0.6655	−0.8938	0–45	6.4
Ice VII	4.898	8.732	−24.99	3–128	3.0
MgO	5.379	1.666	−1.192	0–142	0.6
MgSiO_3_	2.437	0.2434	−0.04751	0–265	1.2
Ar	2.682	18.55	−876.3	0–2	2.5
Kr	2.198	12.04	−459.7	0–2	2.2
Xe	1.728	6.493	−175.2	0–2	1.6
H_2_	3.323	104.0	−7594	0–2.6	3.0 (*P* > 0.34) 26 (*P* < 0.34)
D_2_	3.552	99.32	−7221	0–2.6	2.0 (*P* > 0.15) 18(*P* < 0.15)
(b) Non-solid-state condensed matter (*P* in bar, and *V* in Å^3^)					
PCL	1.03976	6.290	−7.651	0–2000	0.3
H_2_O, 15 °C	0.03343	5.222 × 10^−3^	−1.073 × 10^−4^	0–1000	0
H_2_O, 25 °C	0.03336	5.026 × 10^−3^	−9.835 × 10^−5^	0–1000	0
H_2_O, 35 °C	0.03326	4.901 × 10^−3^	−9.451 × 10^−5^	0–1000	0

The range of the pressure *P* used for each fitting analysis and the maximum % error found in the pressure range are also given^a^, ^b^.

^a^The *P*-vs-*V* data points of the metastable S-III phase between 3–58 GPa (obtained by quenching) reported in ref. 15 were not included in the *P*-vs-*PV* plot. However, including them does not change the quality of the fitting analysis.

^b^Unless mentioned otherwise, the maximum % error refers to the entire pressure region studied.

**Table 3:** The coefficients of the bulk modulus formulas, *B*(*P*) = *B*_0_ + *B*_1_ *P* + *B*_2_ *P* ^2^ + B_3_ *P* 3, obtained for various condensed matter using *P* and *B*(*P*) in GPa units.

	*B*_0,expt_	*B*_0,calc_	*B*_0_	*B*_1_	*B*_2_	*B*_3_
Te	24 (P = 2)^14^	37.68	62.48	4.135	−7.200 × 10^−3^	2.278 × 10^−5^
Se	48.1 (P = 7.7)^18^	28.09	46.99	5.311	−4.021 × 10^−2^	1.516 × 10^−4^
S	14.5^21^	20.10	25.28	2.575	−2.635 × 10^−4^	2.837 × 10^−5^
Sn	54.6°, 55.4°, 54.92^p^	64.31	59.58	5.262	−4.100 × 10^−2^	2.461 × 10^−4^
Au	166.65^6^^,d^	192.09 (P = 4.42)	171.09	3.516	2.146 × 10^−2^	−2.613 × 10^−5^
Cu	133^6^^,q^	184.48 (P = 7.2)	156.32	4.025	−4.760 × 10^−3^	6.803 × 10^−5^
LiF	66.4^6^^,q^	57.94	58.06	4.136	1.520 × 10^−2^	4.610 × 10^−3^
NaF	46.1^6^^,q^	53.20	52.44	5.685	−0.1005	3.900 × 10^−3^
NaCl	23.5^6^	25.20 (P = 0.106)	24.76	4.440	−8.684 × 10^−2^	2.592 × 10^−2^
CsCl	16.8^6^^,q^	27.57	28.79	4.896	−7.036 × 10^−2^	1.290 × 10^−3^
Ice VII	23.9^6^^,q^	38.71 (P = 3.16)	30.37	2.980	−1.950 × 10^−3^	7.809 × 10^−5^
MgO	153–182^30^	170.66	171.63	3.562	−4.270 × 10^−3^	2.269 × 10^−5^
MgSiO_3_	200–340^41^	253.84	255.85	2.995	1.370 × 10^−3^	2.150 × 10^−6^
Ar	2.35^6^^,n^	3.49	3.482	6.053	−1.732	0.9499
Kr	2.49^6^	3.66	3.658	5.909	−1.514	0.8780
Xe	3.02^6^	4.52 (P = 0.05)	4.218	5.942	−1.373	0.7384
H_2_	0.170^51^, 0.174^52^	0.44	0.5707	5.002	−1.521	0.5286
D_2_	0.315^51^, 0.337^52^	0.70	0.7980	4.979	−1.427	0.4953
PCL	3.01^45^	1.683	1.679	8.491	−7.773	86.34
H_2_O (15 °C)	2.140^46^	2.138	2.138	5.627	2.734	4.710
H_2_O (25 °C)	2.210^46^	2.213	2.213	5.605	2.388	5.164
H_2_O (35 °C)	2.250^46^	2.256	2.256	5.632	2.277	5.649

For comparison, the *B*_0,expt_ and *B*_0,calc_ values are also listed (see the text for the definition)^a^, ^b^.

^a^For the references d, l, and n–q, see Section 3 of the SI.

^b^Unless otherwise stated, the *B*_0,expt_ and *B*_0,calc_ refer to the values at *P* = 0. When these values are obtained at a nonzero *P*, the value of *P* is specified in the parenthesis.
