# Erratum: Condensed-matter equation of states covering a wide region of pressure studied experimentally

**DOI:** 10.1038/srep40836

**Published:** 2017-01-12

**Authors:** Elijah E. Gordon, Jürgen Köhler, Myung-Hwan Whangbo

Scientific Reports 6: Article number: 3921210.1038/srep39212; published online: 12 15 2016; updated: 01 12 2017

The original version of this Article contained typographical errors.

In the Abstract,

“Here we show that the simple empirical EOS, P = α^1^(PV) +α^2^(PV)^2^ + α^3^(PV)^3^, in which the pressure P is indirectly related to the volume V through a cubic polynomial of the energy term PV with three fitting parameters α^1^ − α^3^, provides accurate descriptions for the P-vs-V data of condensed matter in a wide region of pressure studied experimentally even in the presence of phase transitions”.

now reads:

“Here we show that the simple empirical EOS, P = α_1_(PV) + α_2_(PV)^2^ + α_3_(PV)^3^, in which the pressure P is indirectly related to the volume V through a cubic polynomial of the energy term PV with three fitting parameters α_1_ − α_3_, provides accurate descriptions for the P-vs-V data of condensed matter in a wide region of pressure studied experimentally even in the presence of phase transitions”.

In the Results section under subheading ‘Applicability to other condensed matter’,

“To establish this point, we examine the experimental *P*-vs-*V* data for various solid-state condensed matter listed in Table 1, which include the elemental Sn, the transition-metals Au and Cu, the alkali halides LiF, NaF, NaCl and CsCl, ice VII, the oxides MgO and MgSiO_3_, the noble gases Ar, Kr and Xe, as well as molecular hydrogen H_2_D_2_”.

now reads:

“To establish this point, we examine the experimental *P*-vs-*V* data for various solid-state condensed matter listed in Table 1, which include the elemental Sn, the transition-metals Au and Cu, the alkali halides LiF, NaF, NaCl and CsCl, ice VII, the oxides MgO and MgSiO_3_, the noble gases Ar, Kr and Xe, as well as molecular hydrogen H_2_ and D_2_”.

These errors have now been corrected in the PDF and HTML versions of the Article.

