# Supplementary material for: Condensed-matter equation of states covering a wide region of pressure studied experimentally
Source: Sci Rep. 2016 Dec 15;6:39212. doi: 10.1038/srep39212 (PMC5156938; doi:10.1038/srep39212)
Supplement: Supplementary Information [file srep39212-s1.pdf]

Supplementary Information

for

Condensed-matter equation of states covering a wide region of pressure studied experimentally

Elijah E. Gordon[1], Jürgen Köhler[2] & Myung-Hwan Whangbo[1,*]

[1] Department of Chemistry, North Carolina State University, Raleigh, NC 27695-8204, USA

[2] Max-Planck-Institut für Festkörperforschung, D-70569 Stuttgart, Germany

* Corresponding author, email: mike_whangbo@ncsu.edu

1. Results of the DFT calculations leading to the EOS, Eq. 1

(a) DFT calculations for the structures of Te under various pressures

The space groups of the known atomic structures of elemental Te at various pressures *P* and temperature *T*, their volumes *V* per atom, the calculated energies *E* per atom as well as the energy *PV*

***P* (GPa)**	***T***	**Space Group (#)**	***E*/Atom (eV)**	***V*/Atom (Å^3)**	***PV*/Atom (eV)**
0.0001	298 K	P3(1)21 (152)[1]	-3.13	33.94	0.00
4.5	298 K	P2(1) (4)[2]	-2.91	27.76	0.78
8	298 K	Cm (12)[3]	-2.91	26.49	1.32
23	473 K	R-3m (166)[4]	-2.51	22.50	3.23
33	298 K	Im-3m (229)[5]	-2.15	20.65	4.25

References

1. Adenis, C.; Langer, V.; Lindqvist, O. *Acta Crystallogr. C* **1989**, 45, 941-942.

2. Aoki, K.; Shimomura, O.; Minomura, S. *J. Phys. Soc. Jpn.* **1980**, 48, 551-556.

3. Takumi, M.; Masamitsu, T.; Nagata, K. *J. Phys.- Condens. Mat.* **2002**, 14, 10609-10613.

4. Hejny, C.; Falconi, S.; Lundegaard, L. F.; McMahon, M. I. *Phys. Rev. B* **2006**, 74, 174119.

5. Parthasarathy, G.; Holzapfel, W. B. *Phys. Rev. B* **1988**, 37, 8499-8501.

(b) DFT calculations for the structures of Se under various pressures

The space groups of the known atomic structures of elemental Se at various pressures *P* and temperature *T*, their volumes *V* per atom, the calculated energies *E* per atom as well as the energy *PV*

P **(GPa)**	***T***	**Space Group (#)**	***E*****/Atom (eV)**	***V*****/Atom (Å^3)**	***PV*****/Atom (eV)**
0.0001	298 K	P2(1)/c (14)[1]	-3.47	30.13	0.00
4.6	298 K	P3(1)21 (152)[2]	-3.30	22.67	0.65
23	298 K	P2(1) (4)[3]	-2.88	17.37	2.49
87.9	298 K	R-3m (166)[3]	-1.53	12.93	7.09
140	298 K	Im-3m (229)[3]	-0.22	11.25	9.83

References

1. Marsh, R. E.; Pauling, L.; McCullough, J. D. *Acta Crystallogr.* **1953**, 6, 71.

2. Parthasarathy, G.; Holzapfel, W. B. *Phys. Rev. B* **1988**, 38, 10105.

3. Akahama, Y.; Kobayashi, M.; Kawamura, H. *Phys. Rev. B* **1993**, 47, 20-26.

(c) DFT calculations for the structures of S under various pressures

The space groups of the known atomic structures of elemental S at various pressures *P* and temperature *T*, their volumes *V* per atom, the calculated energies *E* per atom as well as the energy *PV*

P **(GPa)**	***T***	**Space Group (#)**	***E*****/Atom (eV)**	***V*****/Atom (Å^3)**	***PV*****/Atom (eV)**
0.0001	298 K	Fddd (70)[1]	-4.07	25.97	0.00
3	673 K	P3(2)21 (154)[2]	-4.00	20.81	0.39
12	298 K	I4(1)/acd (142)[3]	-3.78	16.7	1.25
160	298 K	R-3m (166)[4]	-1.07	8.77[a] (8.87)	8.85
173	298 K	R-3m (166)[5]	-0.76	8.50[a] (10.68)	9.18
206.5	298 K	R-3m (166)[6]	-0.20	8.01	10.32

[a] We employed the volumes optimized by DFT calculations, because the experimental value at 173 GPa should not be greater than that at 160 GPa (the numbers in the parentheses).

References

1. Warren, B. E.; Burwell, J. T. *J. Chem. Phys.* **1935**, 3, 6-8.

2. Crichton, W. A.; Vaughan, G. B. M.; Mezouar, M., *Z. Kristallogr.* **2001**, 216, 417-419.

3. Degtyareva, O.; Gregoryanz, E.; Somayazulu, M.; Dera, P.; Mao, H.-K.; Hemley, R. J., *Nat. Mater.* **2005**, 4, 152-155.

4. Degtyareva, O.; Gregoryanz, E.; Somayazulu, M.; Dera, P.; Mao, H.-K.; Hemley, R. J., *Phys. Rev. B* **2005**, 71, 214104.

5. Einaga, M.; Sakata, M.; Ishikawa, T.; Shimizu, K.; Eremets, M. I.; Drozdov, A. P.; Troyan, I. A.; Hirao, N.; Ohishi, Y. arXiv: 1509.03156v1 **2015**

6. Luo, H.; Greene, G.; Ruoff, A. L. *Phys. Rev. Lett.* **1993**, 71, 2943-2946.

(d) DOS plots calculated for Te under various pressures

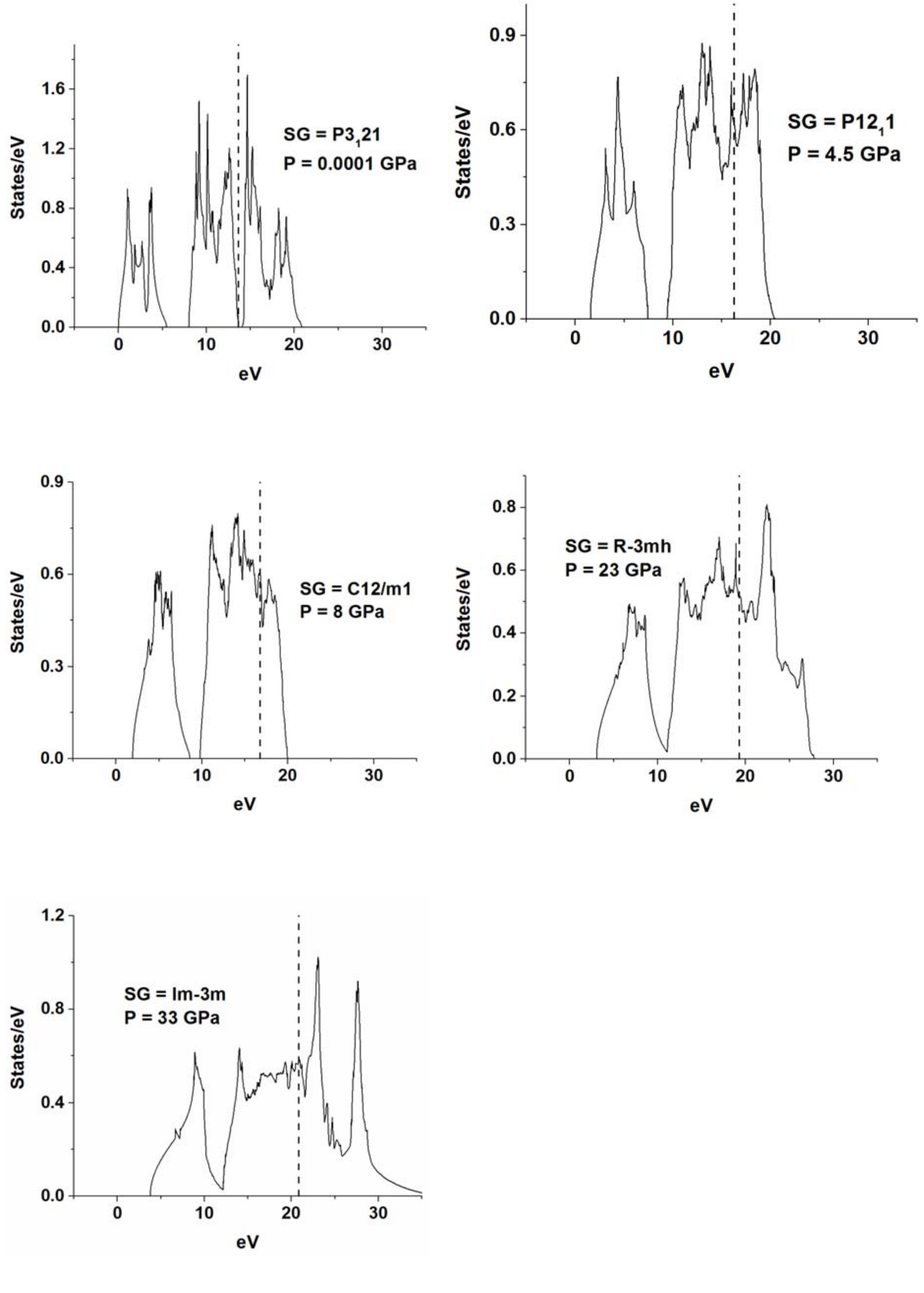

(e) DOS plots calculated for Se under various pressures

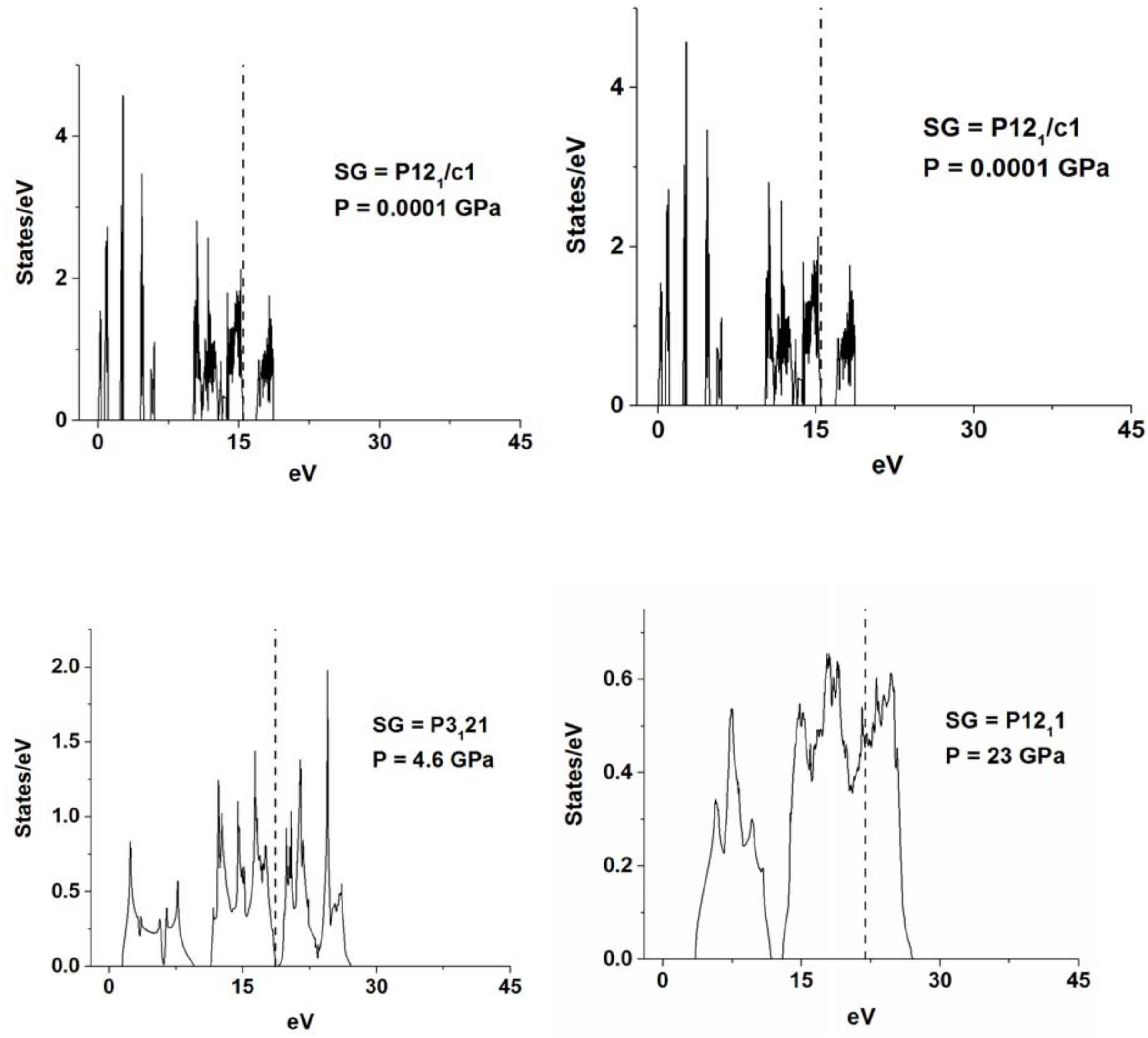

(f) DOS plots calculated for S under various pressures

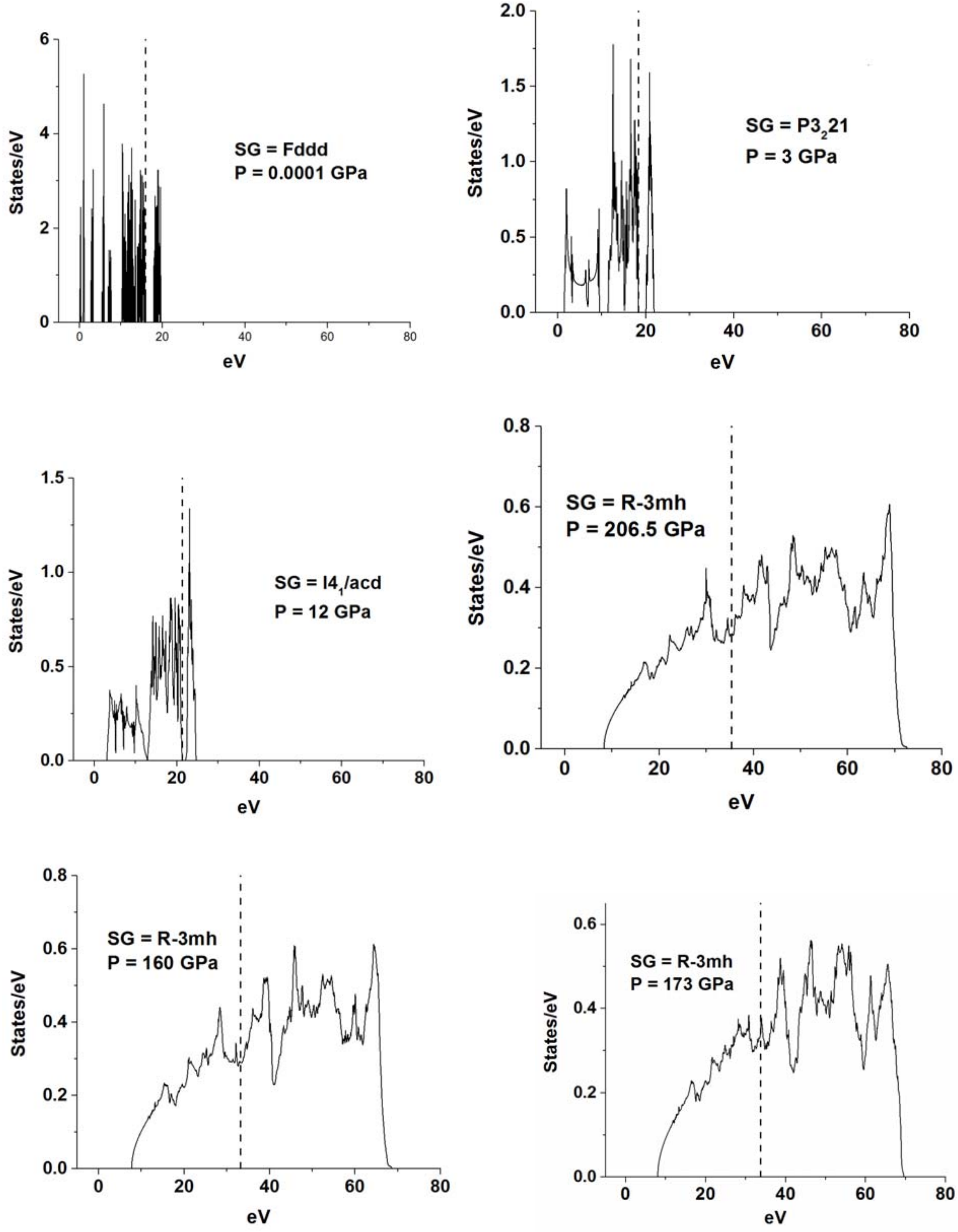

2. Analyses of the empirical EOSs for chalcogen

(a) Energy-vs-*P*, *P*-vs-*PV*, % error-vs-*P*, and *B*(*P*)-vs-*P* plots obtained for Se

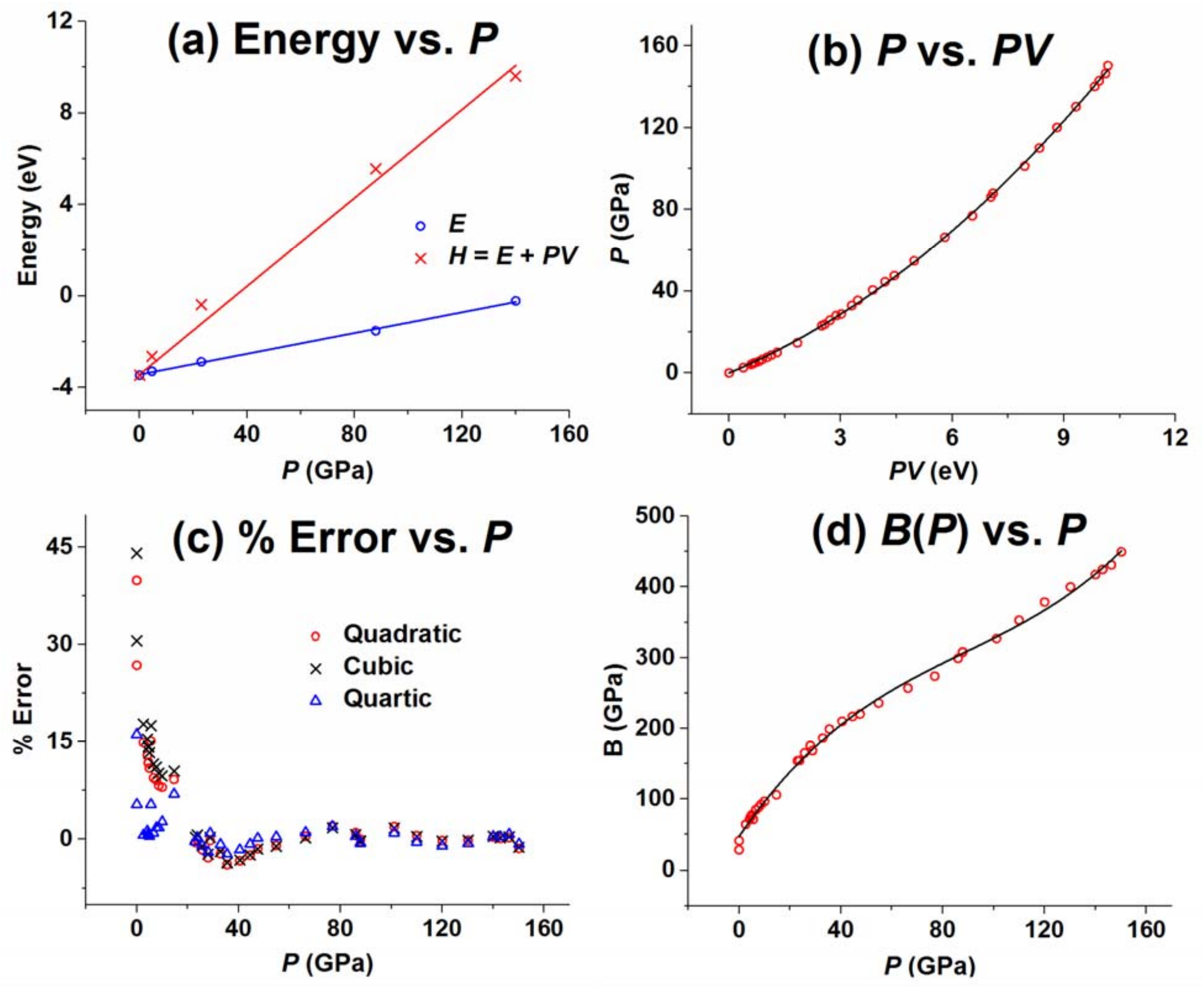

a) The E vs. P and H vs. P plots calculated for Se, where E, H and PV are in units of eV. The fitting coefficients a_1, a_0 and b_1 for $E = a_1P + a_0$ and $H = b_1P + a_0$ are -3.2146, 0.0206 and 0.0945, respectively. b) The P vs. PV plot obtained for Se, where the solid line is the fitting curve obtained from the cubic approximation. c) The pressure-dependence of the % error, $100\times(P_{calc} - P_{expt})/P_{expt}$, obtained for Se from the cubic approximation. d) The pressure-dependence of the bulk modulus $B(P)$ calculated for Se.

(b) Energy-vs-*P*, *P*-vs-*PV*, % error-vs-*P*, and *B*(*P*)-vs-*P* plots obtained for S

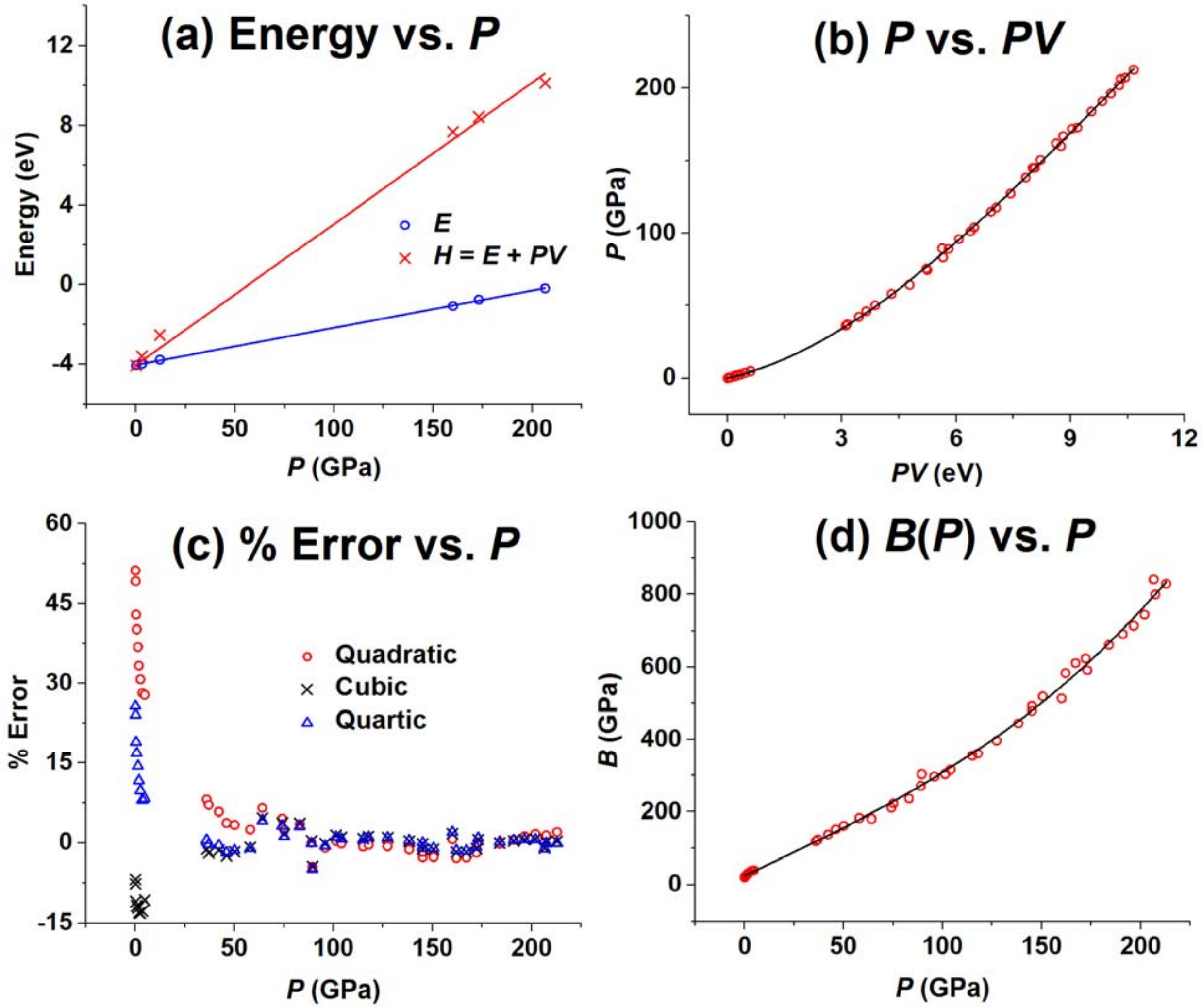

a) The E vs. P and H vs. P plots calculated for S, where E, H and PV are in units of eV. The fitting coefficients a_1, a_0 and b_1 for $E = a_1P + a_0$ and $H = b_1P + a_0$ are -3.8808, 0.0175 and 0.0696, respectively. b) The P vs. PV plot obtained for S, where the solid line is the fitting curve obtained from the cubic approximation. c) The pressure-dependence of the % error, $100\times(P_{calc} - P_{expt})/P_{expt}$, obtained for S from the cubic approximation. d) The pressure-dependence of the bulk modulus $B(P)$ calculated for S.

(c) The fitting of the *P*-vs-*V* data for chalcogens, used for DFT calculations, with the equation $P = \alpha_1(PV) + \alpha_2(PV)^2$

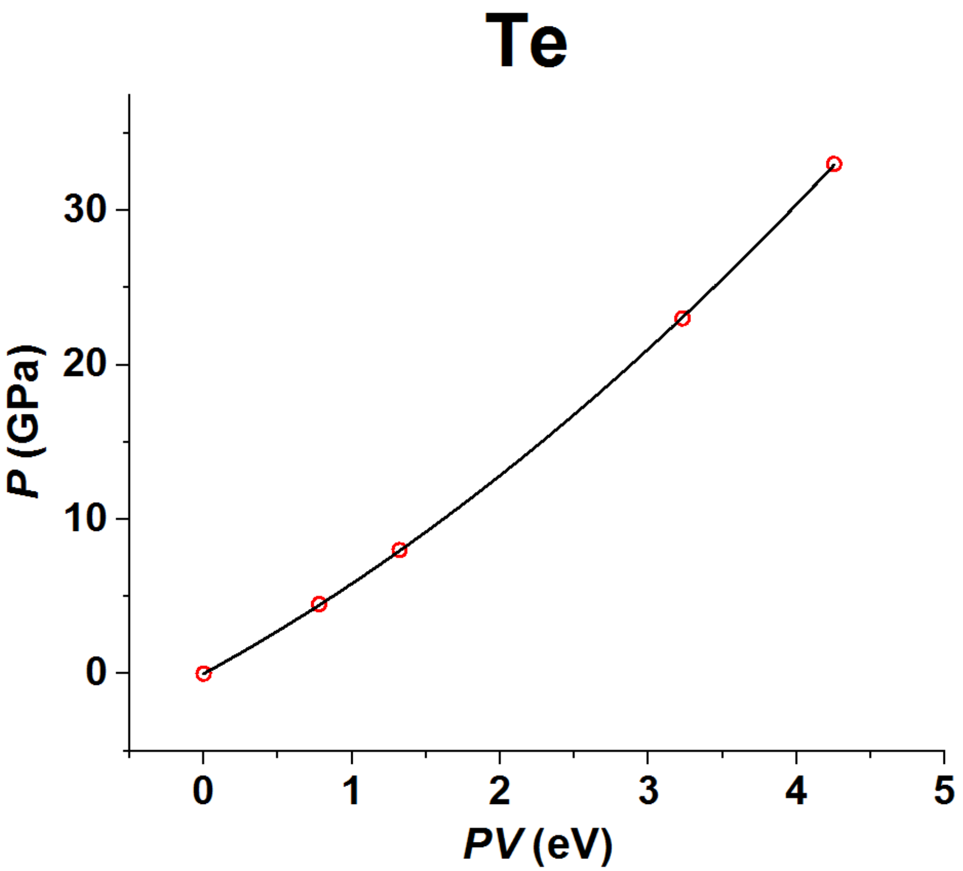

For Te: α_1 = 5.245 and α_2 = 0.5886

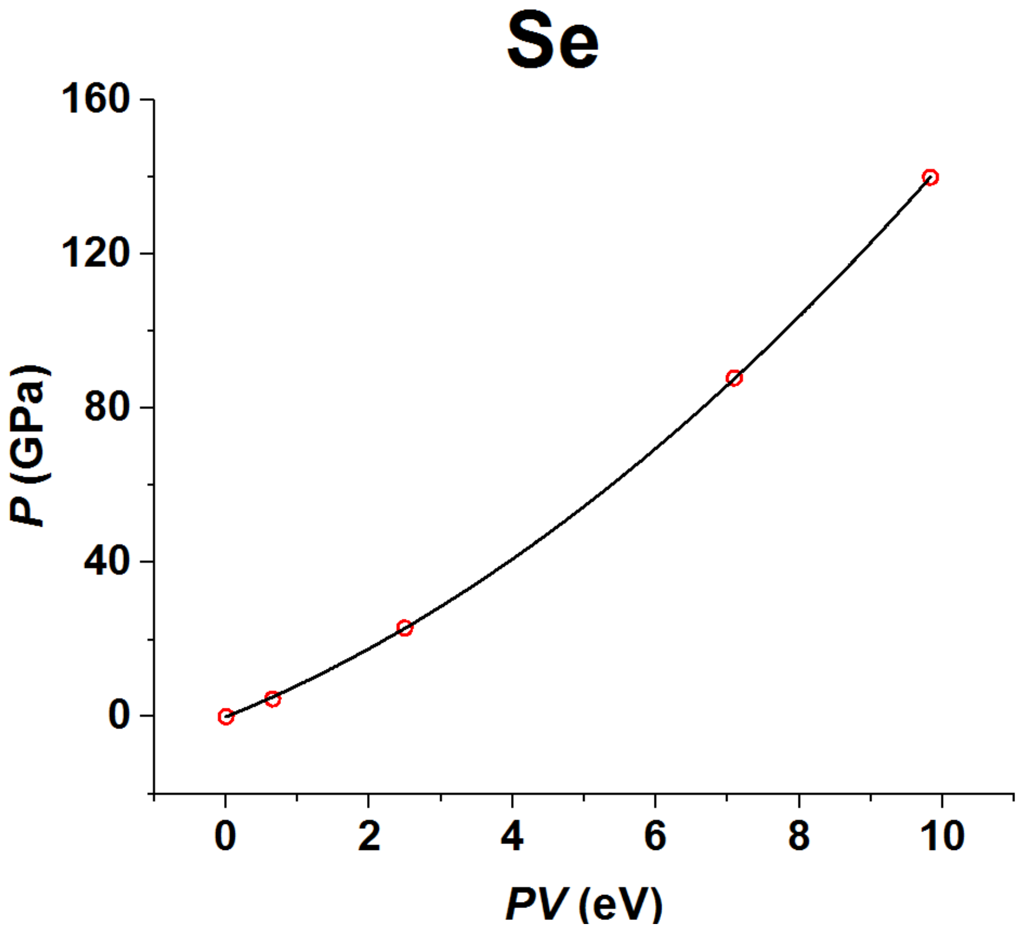

For Se: α_1 = 7.500 and α_2 = 0.6866

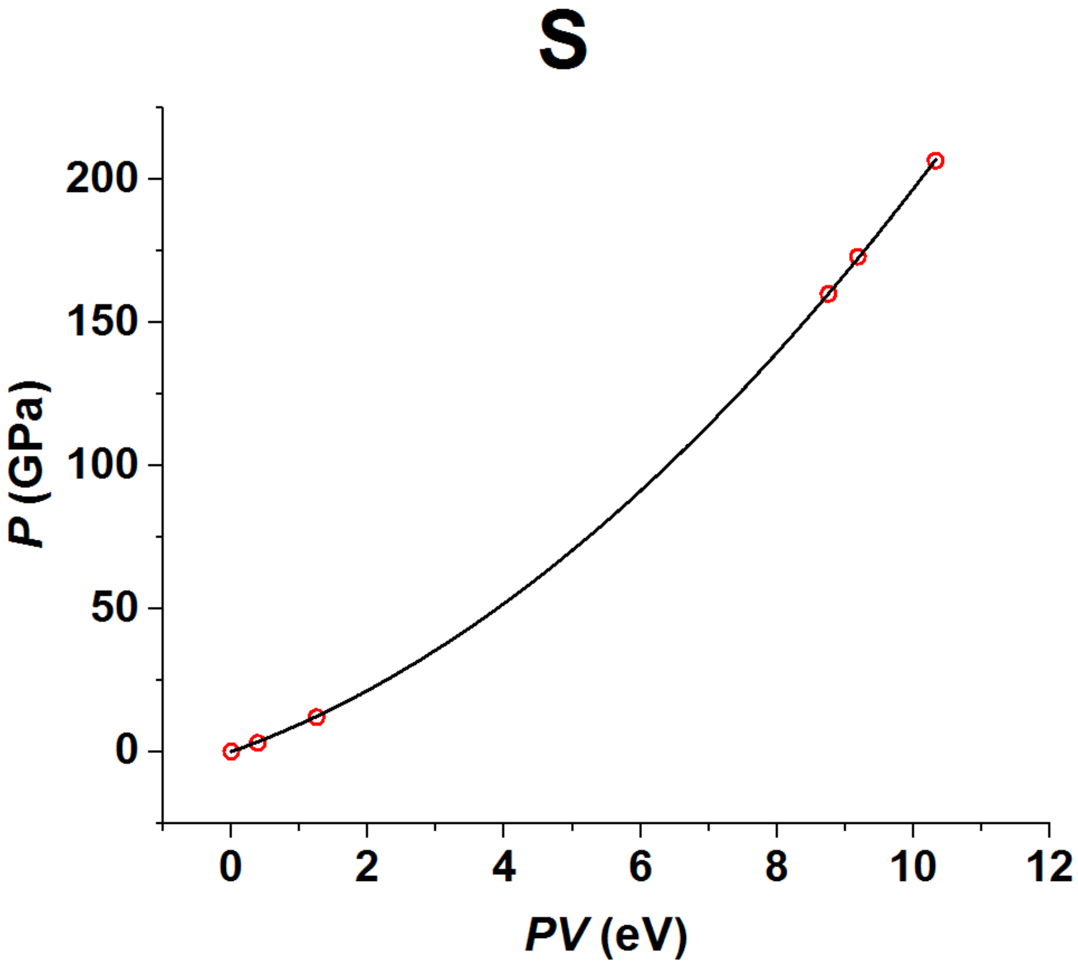

For S: $\alpha_1 = 8.471$ and $\alpha_2 = 1.121$

(d) The coefficients α_i of the isothermal EOS, $P = \alpha_1(PV) + \alpha_2(PV)^2 + \alpha_3(PV)^3 + \ldots$, for elemental chalcogens in the quadratic, cubic and quartic approximations, obtained by using *P* in GPa units and *V* in Å^3 units.

1) For Te from the 0 – 330 GPa region

Approximation	α_1	α_2	α_3	α_4
Quadratic	0.04491	0.9036×10^{-5}		
Cubic	0.03785	1.501×10^{-5}	-1.130×10^{-9}	
Quartic	0.03769	1.527×10^{-5}	-1.244×10^{-9}	1.523×10^{-14}

2) For Se from the 0 – 150 GPa region

Approximation	α_1	α_2	α_3	α_4
Quadratic	0.04644	2.712×10^{-5}		
Cubic	0.04782	2.421×10^{-5}	1.325×10^{-9}	
Quartic	0.03856	5.998×10^{-5}	-37.34×10^{-9}	1.251×10^{-11}

3) For S from the 0 – 213 GPa region

Approximation	α_1	α_2	α_3	α_4
Quadratic	0.05824	4.035×10^{-5}		
Cubic	0.03587	7.987×10^{-5}	-1.620×10^{-8}	
Quartic	0.04840	4.250×10^{-5}	$+1.820\times10^{-8}$	-9.942×10^{-12}

(e) Pressure-dependence of the absolute errors, $\Delta P = P_{calc} - P_{expt}$, calculated for Te, Se and S

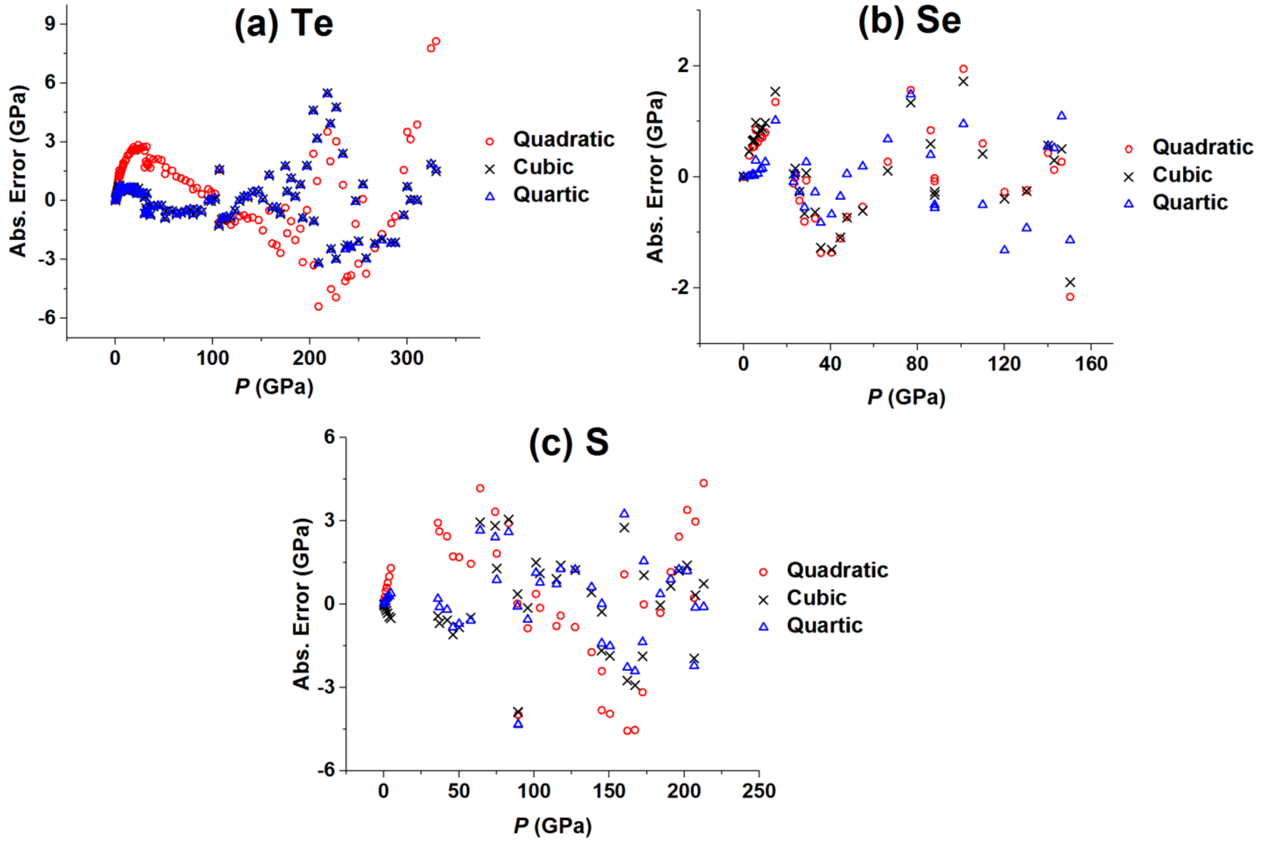

The pressure-dependence of the absolute error, ($P_{calc} - P_{expt}$), obtained for (a) Te, (b) Se and (c) S from the cubic approximation.

3. Analyses of the empirical EOSs obtained for various condensed matter listed in Table 1

In this section we show the P-vs. PV, the % error-vs-P, and the $B(P)$-vs-P plots for the various condensed matter studied in this work. The references (a) – (q) were defined in Tables 1 and 3.

(a) Sn

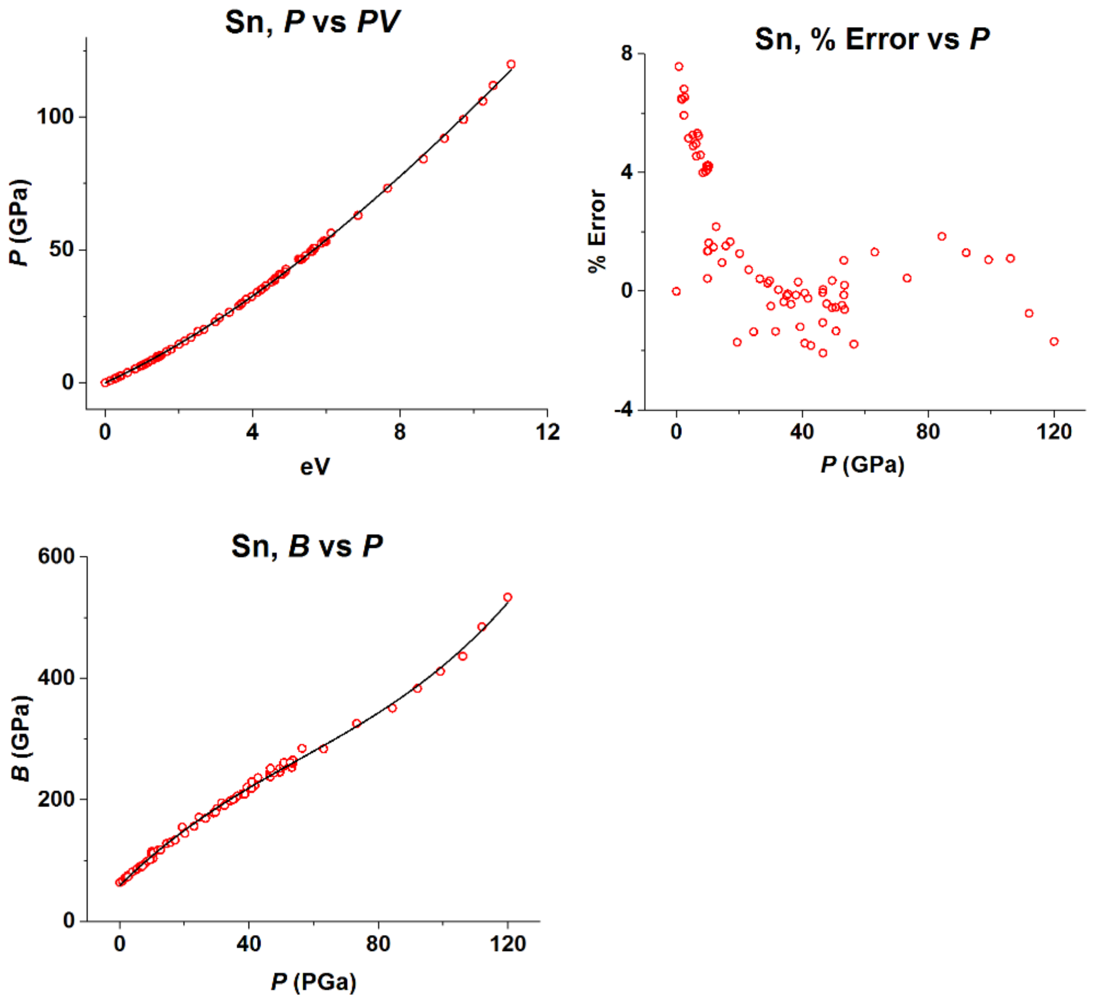

References

(a) Liu, M., & Liu, L., Compressions and phase transitions of tin to half a megabar. *High Temp.-High Press*. **18**, 79-85 (1986)

(b) Olijnyk, H., & Holzapfel, W. B., Phase transitions in Si, Ge and Sn under pressure. *J. Phys.-Paris Colloq*. **45**, Suppl. 11, C8-153 (1984).

(c) Desgreniers, S., Vohra, Y. K., & Ruoff, A. L., Tin at high pressure: An energy-dispersive x-ray diffraction study to 120 GPa. *Phys. Rev. B* **39**, 10359-10361 (1988).

(o) Kamioka, H., Temperature Variations of Elastic Moduli up to Eutectic Temperature in Tin-Bismuth Alloys, *Jpn. J. Appl. Phys*. **22**, 1805 (1983).

(p) Vaiyda, S. N., Kennedy, G. C., Compressibility of 22 elemental solids to 45 KB, *J. Phys. Chem. Solids* **31**, 2329 (1970).

(b) Au

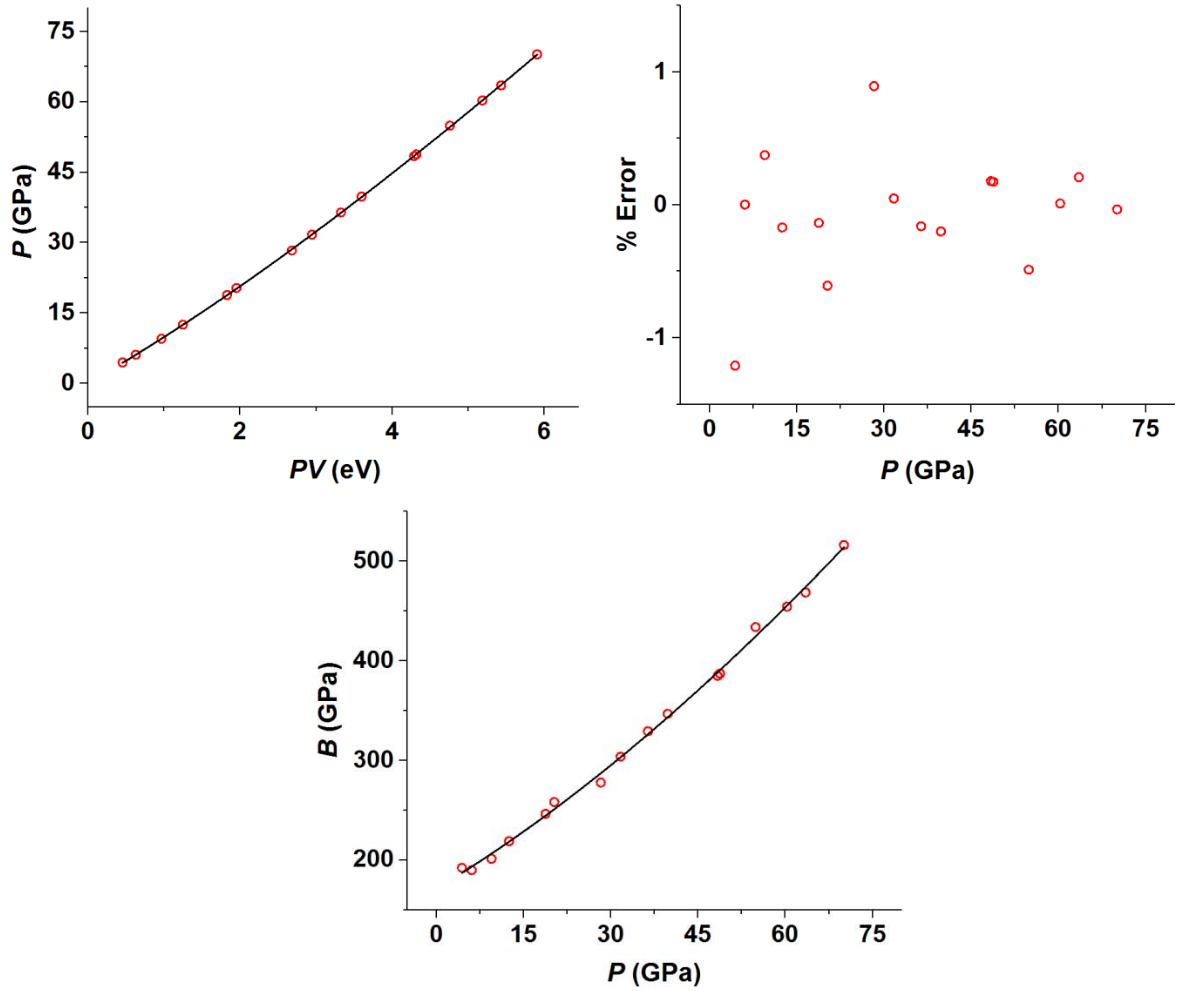

References

(d) Heinz, D. L. & Jeanloz, R., The equation of state of the gold calibration standard. *J. Appl. Phys.* **55**, 885-893 (1984).

(c) Cu

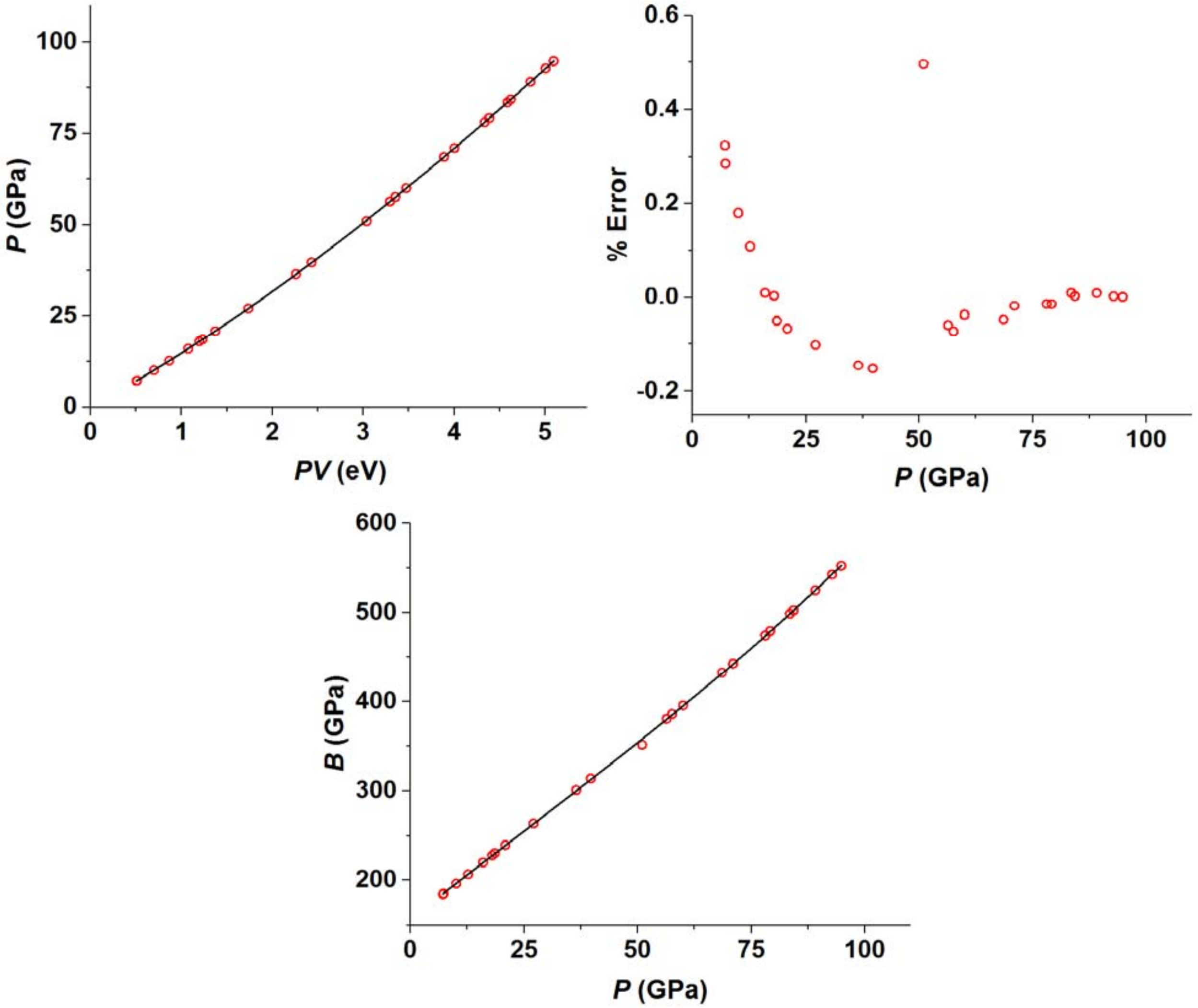

References

(e) Mao, H. K., & Bell, P. M., Specific volume measurements of Cu, Mo, Pd, and Ag and calibration of the ruby R_1 fluorescence pressure gauge from 0.006 to 1 Mbar. *J. Appl. Phys.* 46, 3276-3283 (1978).

(q) Barsch, G. R. & Chang, Z. P., Adiabatic, Isothermal, and Intermediate Pressure Derivatives of the Elastic Constants for Cubic Symmetry. *Phys. Status Solidi B* **19**, 139-151 (1967).

(d) LiF

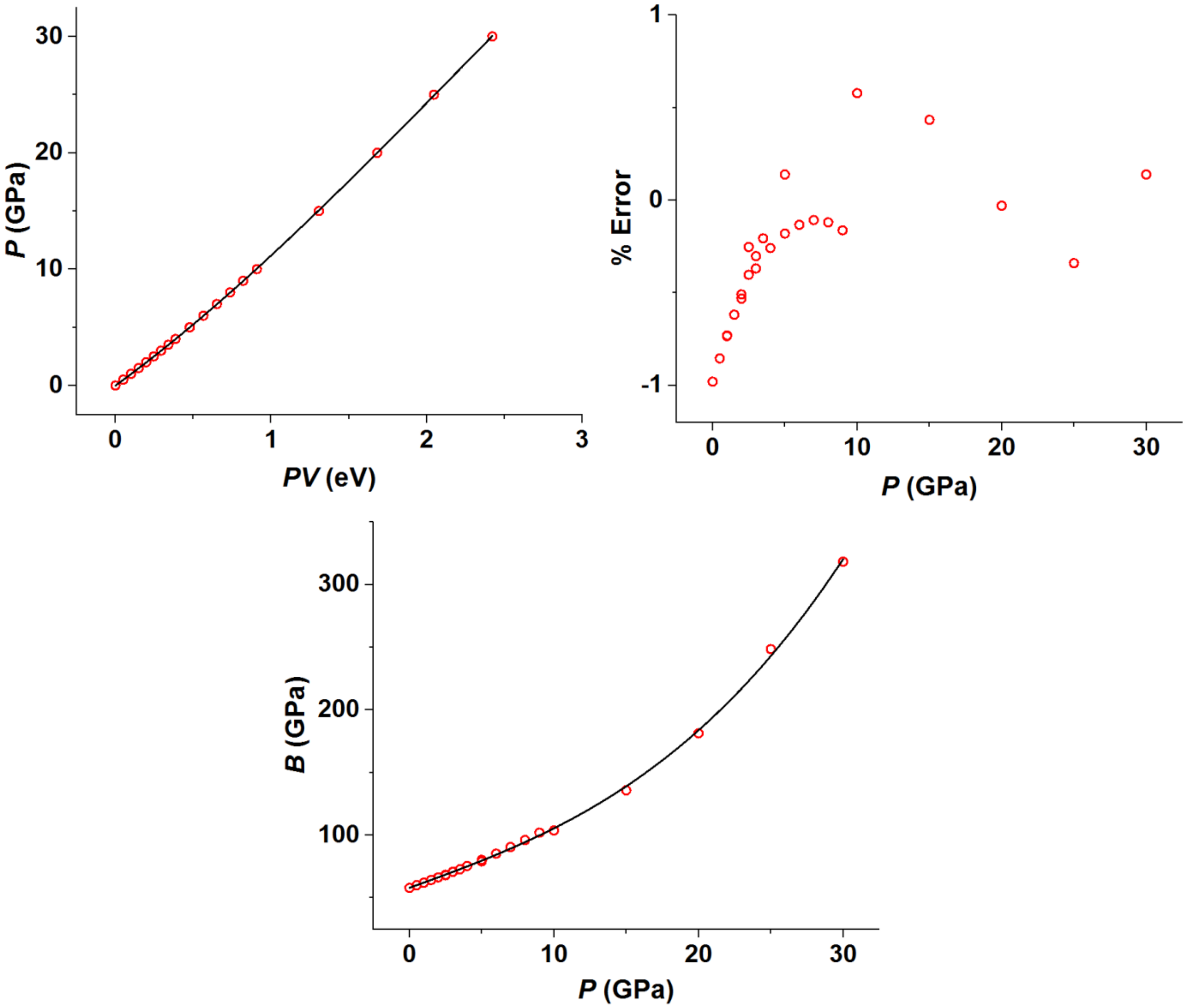

References

(f) Yagi, T. Experimental determination of thermal expansivity of several alkali halides at high pressure. *J. Phys. Chem. Solids* **39**, 563-571 (1978).

(g) Pagannone, M. & Drickamer, H. G., Effect of high pressure on the compressibilities of NaI, LiF, and NaF. *J. Chem. Phys.* **43**, 2266-2268 (1965).

(h) Boehler, R. & Kennedy, G. C., Thermal expansion of LiF at high pressures. *J. Phys. Chem. Solids* **41**, 1019-1022 (1980).

(q) Barsch, G. R. & Chang, Z. P., Adiabatic, Isothermal, and Intermediate Pressure Derivatives of the Elastic Constants for Cubic Symmetry. *Phys. Status Solidi B* **19**, 139-151 (1967).

(e) NaF

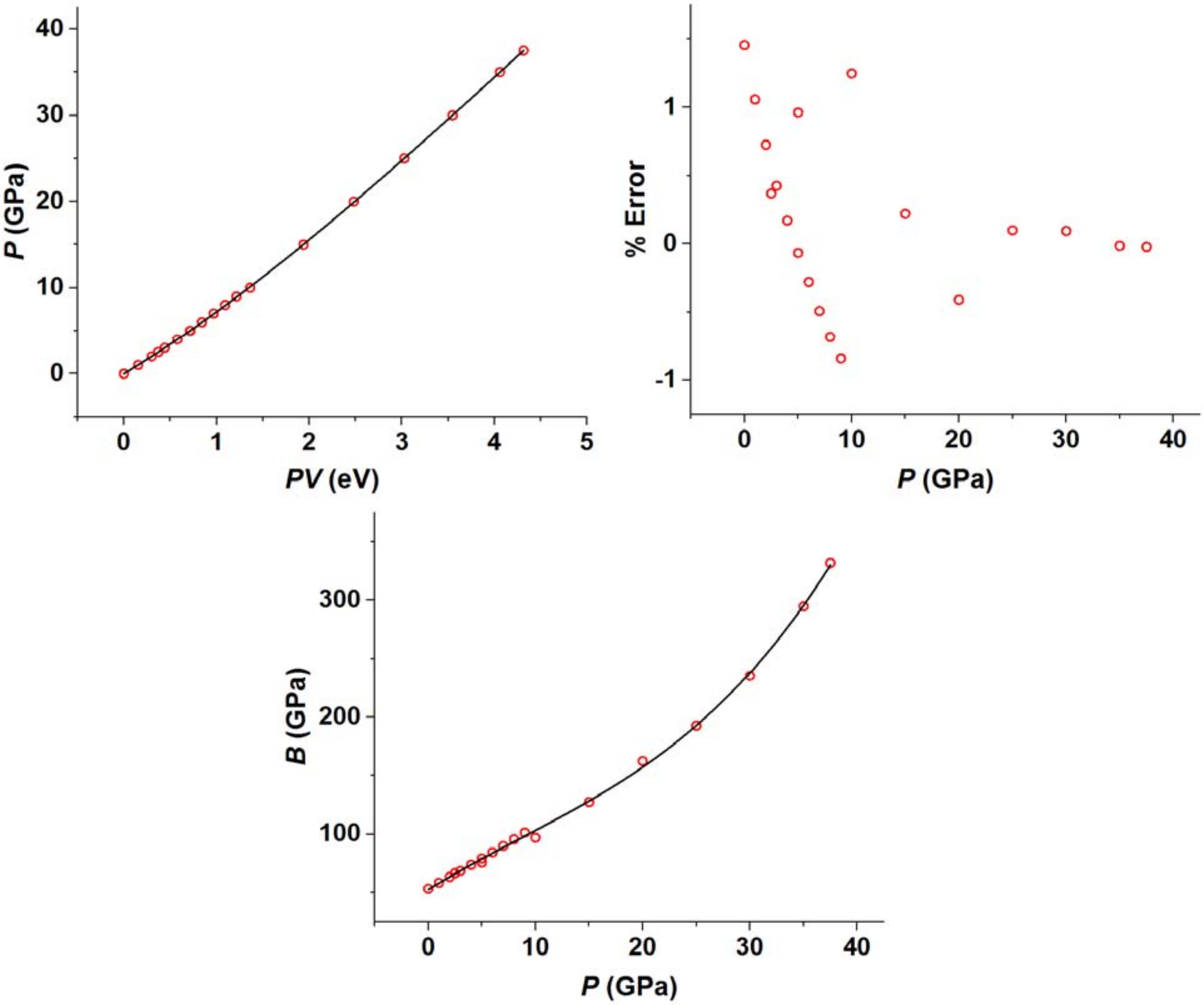

References

(f) Yagi, T. Experimental determination of thermal expansivity of several alkali halides at high pressure. *J. Phys. Chem. Solids* **39**, 563-571 (1978).

(g) Pagannone, M. & Drickamer, H. G., Effect of high pressure on the compressibilities of NaI, LiF, and NaF. *J. Chem. Phys.* **43**, 2266-2268 (1965).

(q) Barsch, G. R. & Chang, Z. P., Adiabatic, Isothermal, and Intermediate Pressure Derivatives of the Elastic Constants for Cubic Symmetry. *Phys. Status Solidi B* **19**, 139-151 (1967).

(f) NaCl

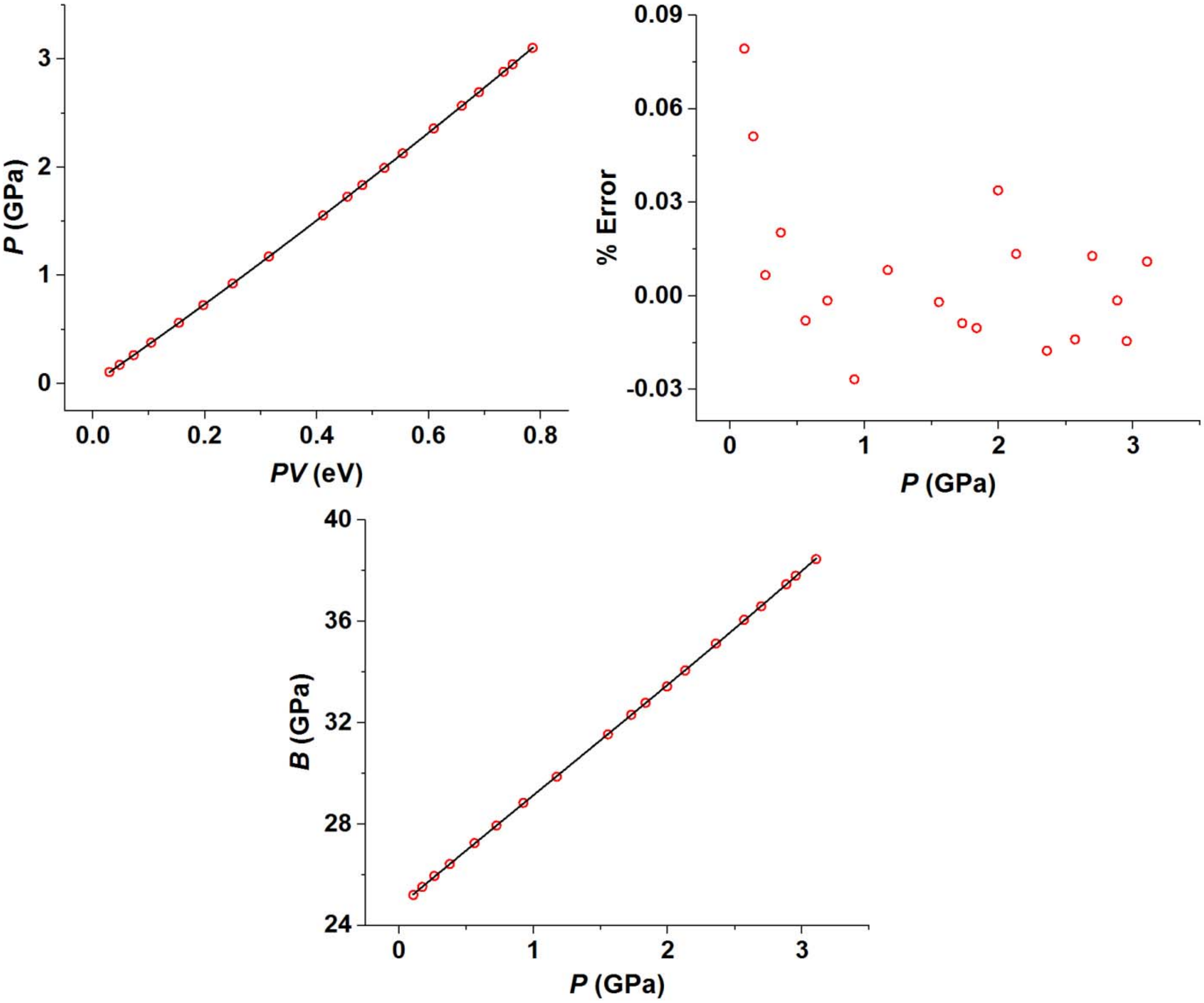

References

(i) Boehler, R., & Kennedy, G. C., Equation of state of sodium chloride. *J. Phys. Chem. Solids* **41**, 517-523 (1980).

(q) Barsch, G. R. & Chang, Z. P., Adiabatic, Isothermal, and Intermediate Pressure Derivatives of the Elastic Constants for Cubic Symmetry. *Phys. Status Solidi B* **19**, 139-151 (1967).

(g) CsCl

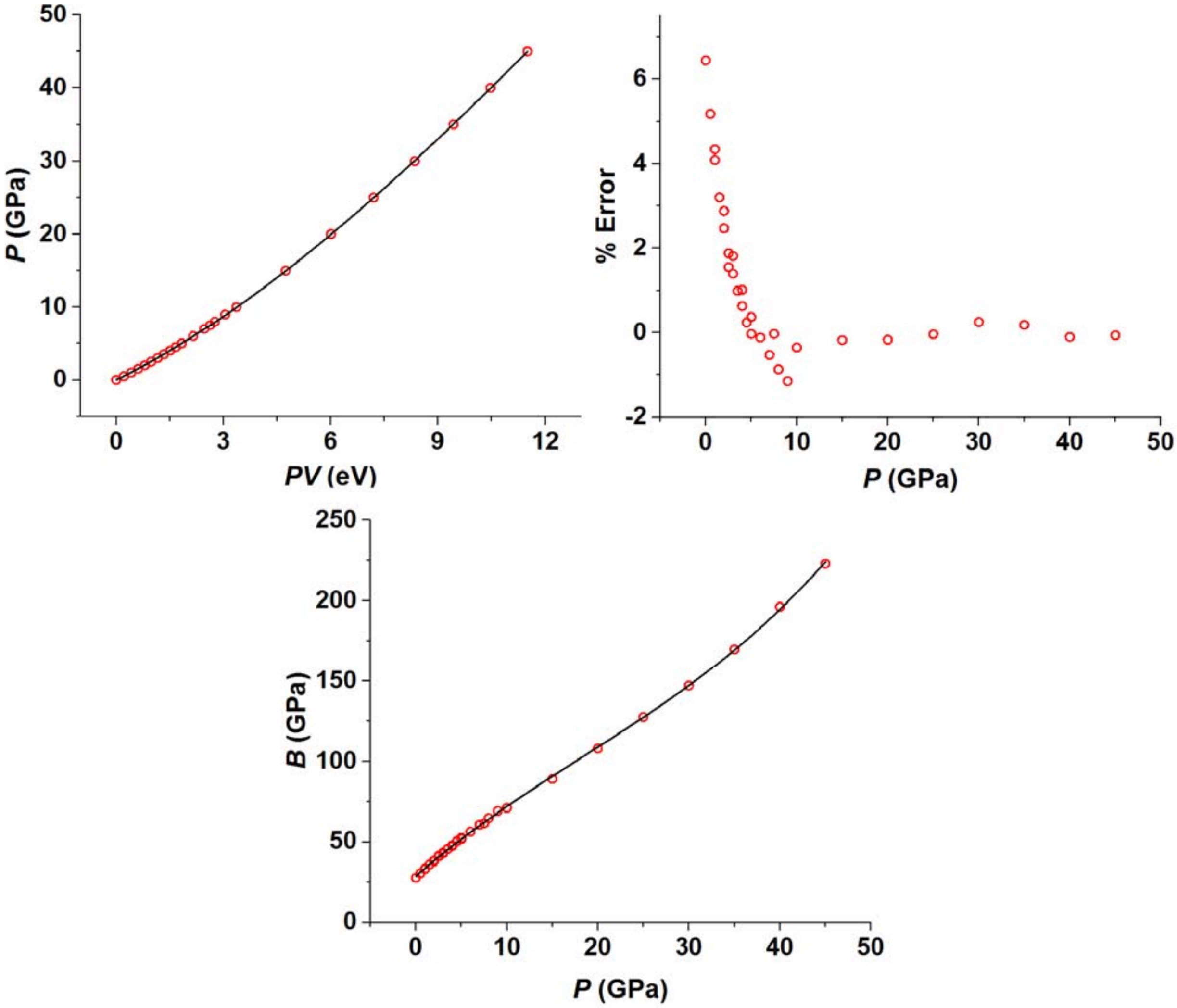

References

(f) Yagi, T. Experimental determination of thermal expansivity of several alkali halides at high pressure. *J. Phys. Chem. Solids* **39**, 563-571 (1978).

(j) Perez-Albuerne, E. A., & Drickamer, H. G., Effect of high pressures on the compressibilities of seven crystals having the NaCl or CsCl structure. *J. Chem. Phys.* **43**, 1381-1387 (1965).

(k) Vaidya, S. N. & Kennedy, G. C., Compressibility of 27 halides to 45 kbar. *J. Phys. Chem. Solids* **32**, 951-964 (1971).

(h) Ice VII

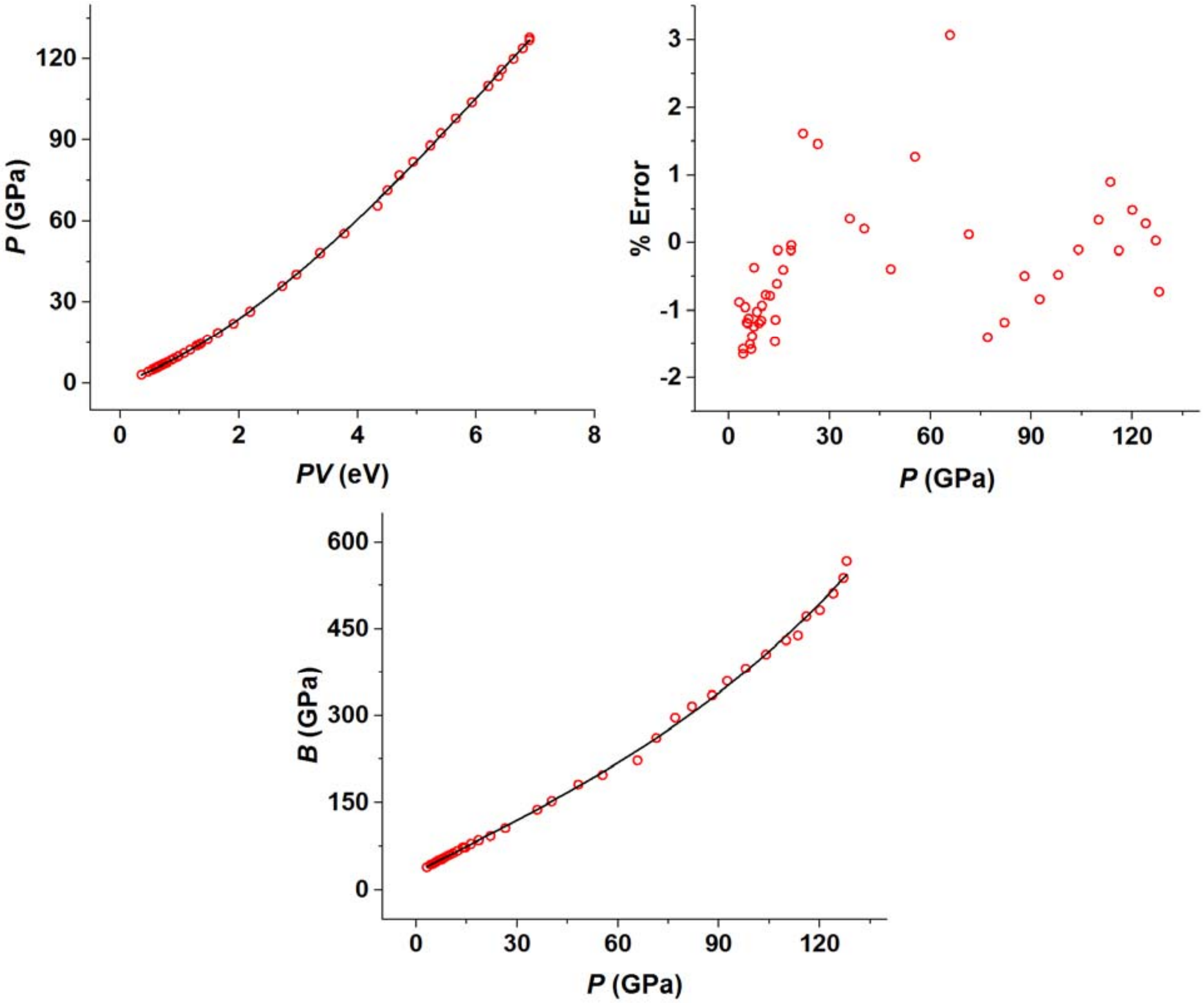

References

(l) Fei, Y., Mao, H. K., & Hemley, R. J. Thermal expansivity, bulk modulus, and melting curve of H_2O-ice VII to 20 GPa. *J. Chem. Phys.* **99**, 5369-5373 (1993).

(m) Hemley, R. J., Jephcoat, A. P., Mao, H. K., Zha, C. S., Finger, L. W., & Cox, D. E. Static compression of H_2O-ice to 128 GPa (1.28 Mbar). *Nature* **330**, 737-740 (1987).

(i) Ar

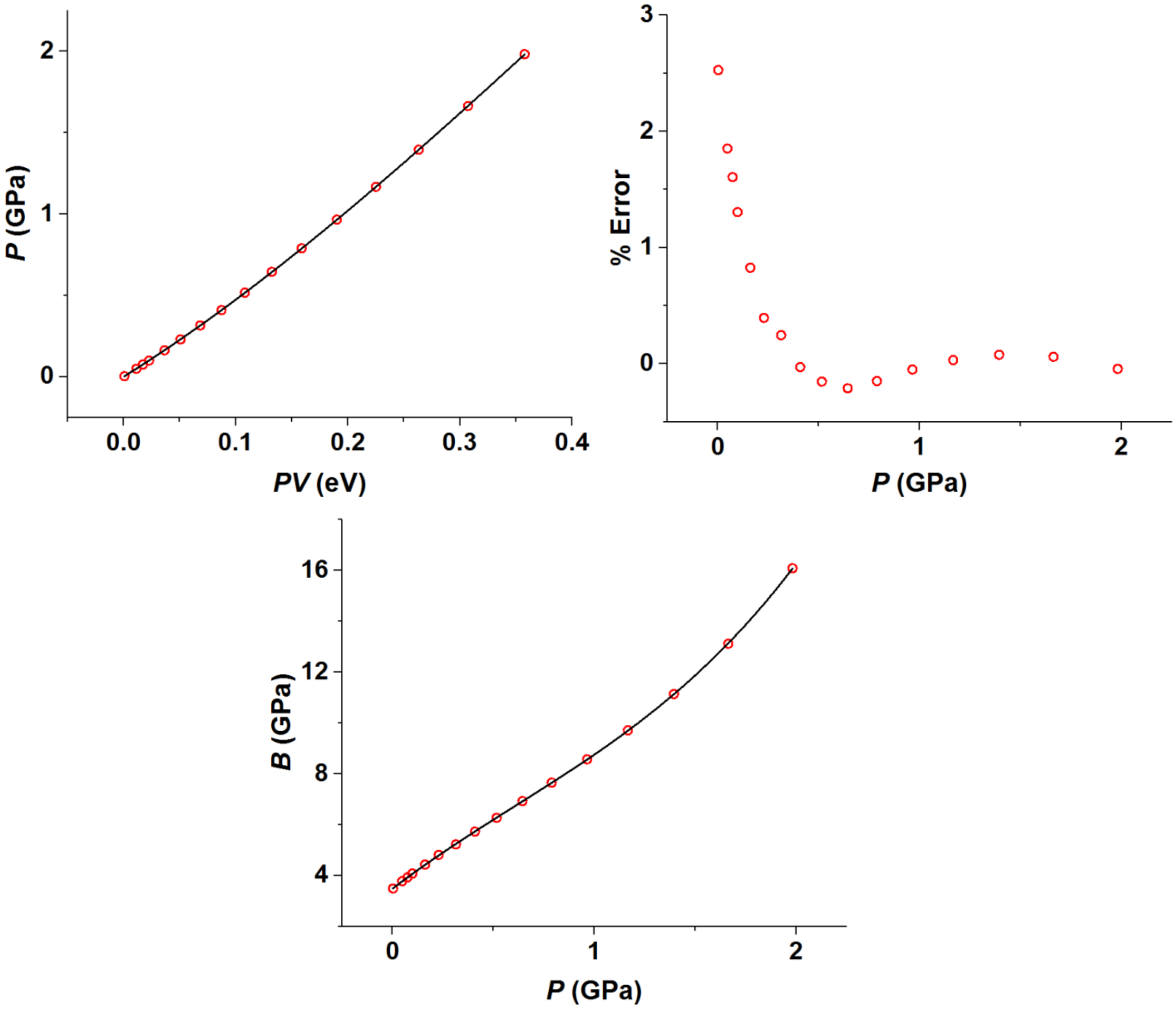

References

(n) Anderson, M. S., & Swenson, C. A., Experimental equations of state for the rare gas solids. *J. Phys. Chem. Solids* **36**, 145-162 (1975).

(j) Kr

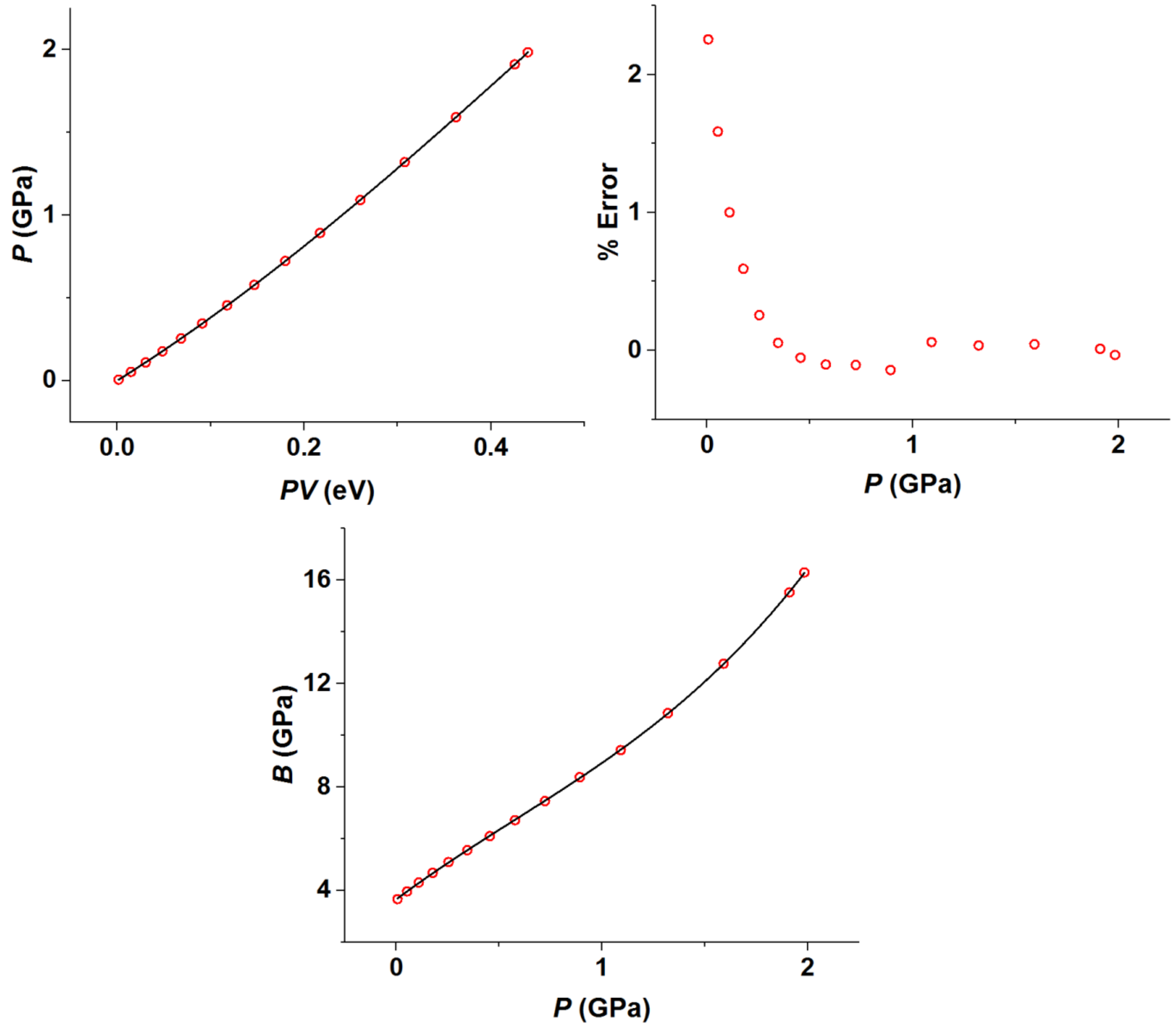

References

(n) Anderson, M. S., & Swenson, C. A., Experimental equations of state for the rare gas solids. *J. Phys. Chem. Solids* **36**, 145-162 (1975).

(k) Xe

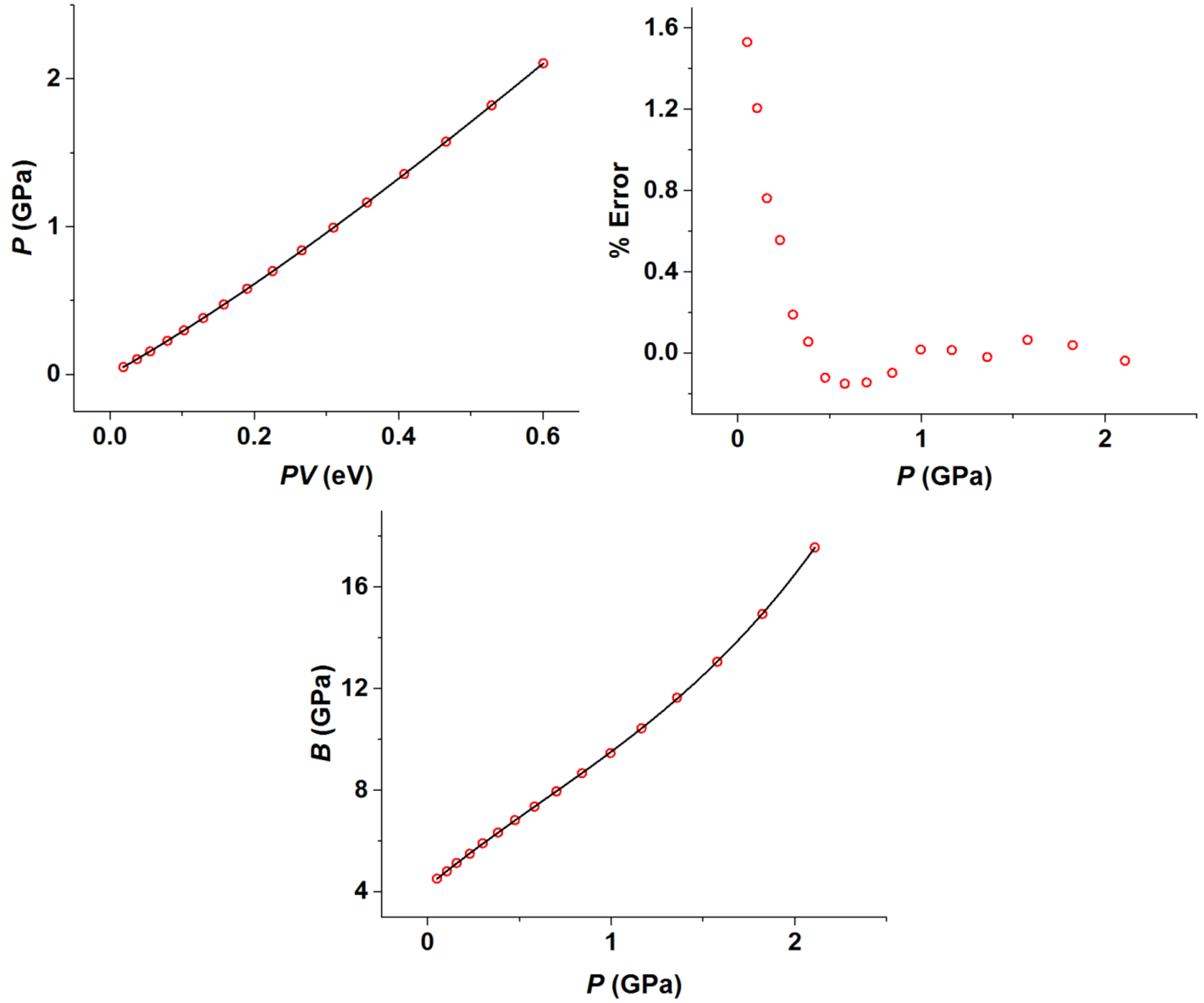

References

(n) Anderson, M. S., & Swenson, C. A., Experimental equations of state for the rare gas solids. *J. Phys. Chem. Solids* **36**, 145-162 (1975).

(k) H_2

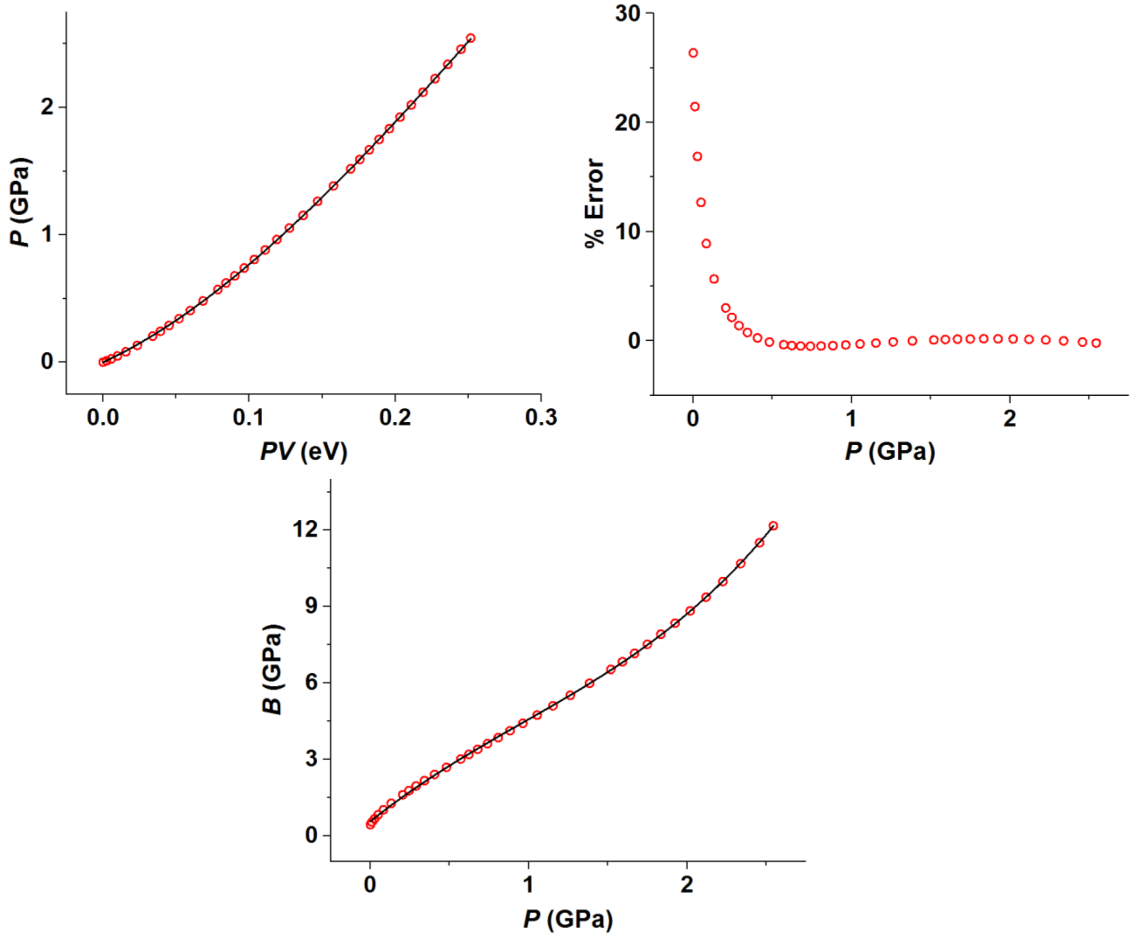

P (GPa)
0
1
2
0.0
0.1
0.2
0.3
PV (eV)
% Error
30
20
10
0
0
1
2
P (GPa)
B (GPa)
12
9
6
3
0
0
1
2
P (GPa)

(l) D_2

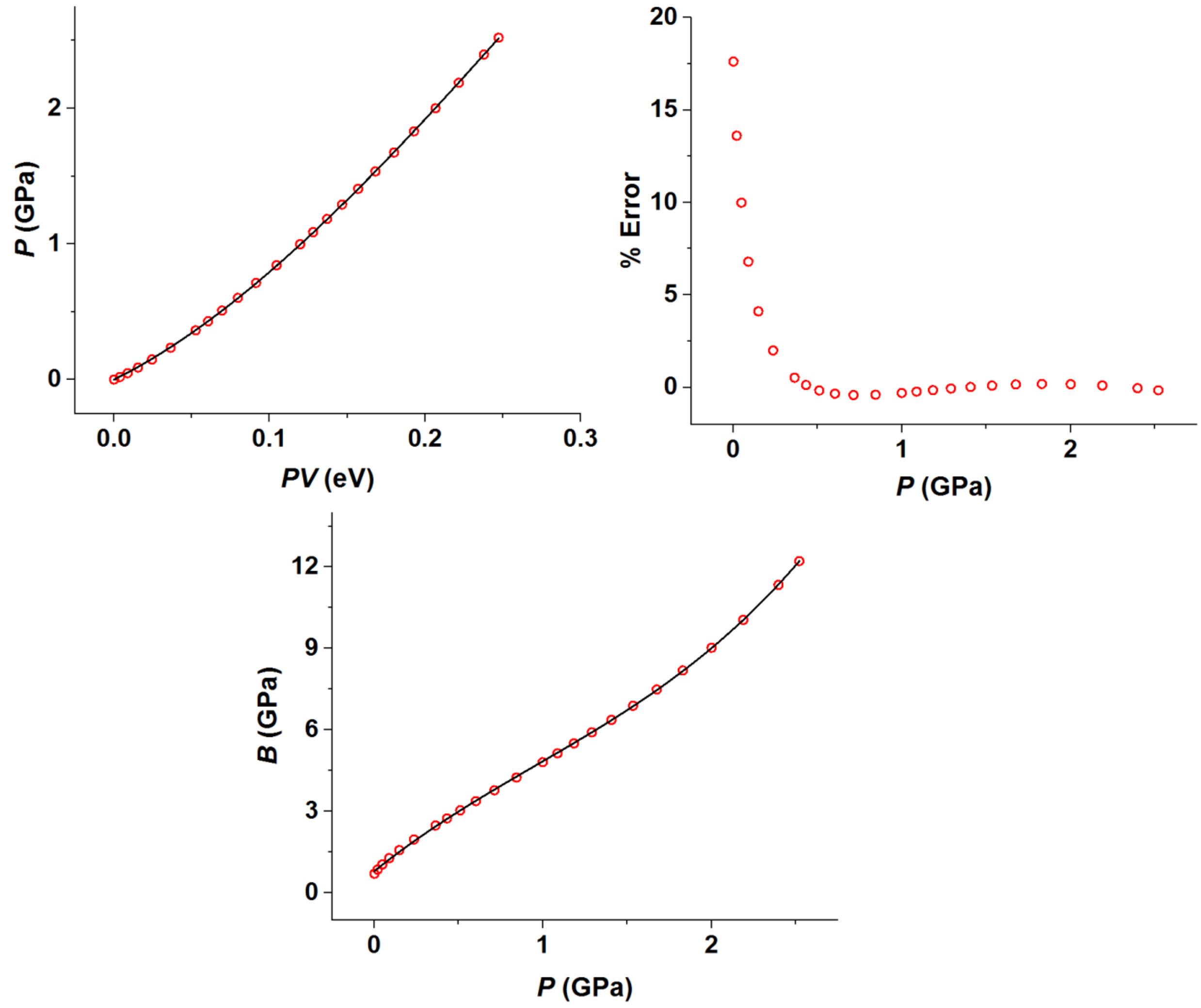

P (GPa)
PV (eV)
0.0
0.1
0.2
0.3
0
1
2
% Error
P (GPa)
20
15
10
5
0
0
1
2
B (GPa)
P (GPa)
12
9
6
3
0
0
1
2

(m) Polymer PCL

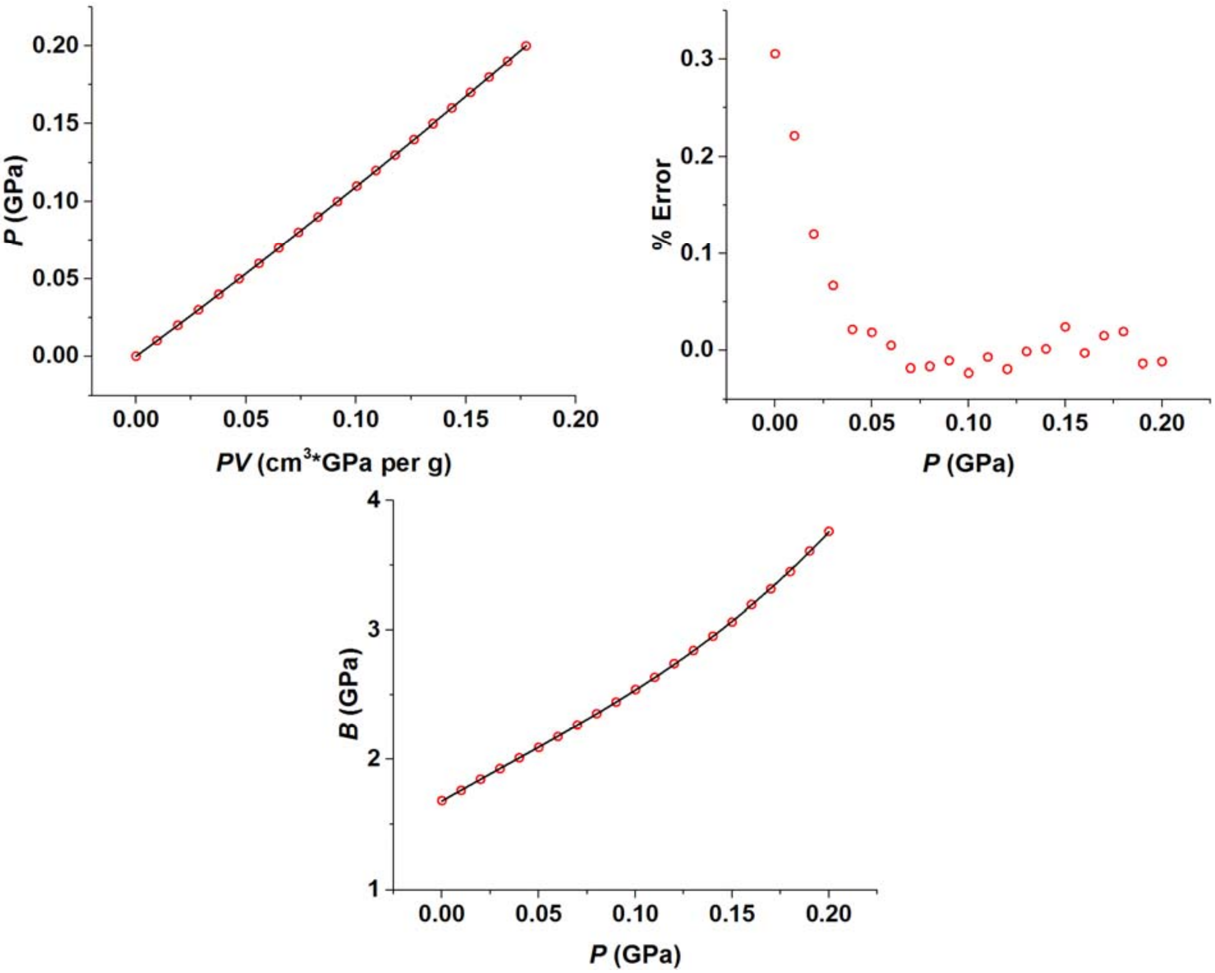

(n) Liquid H_2O at 15 °C

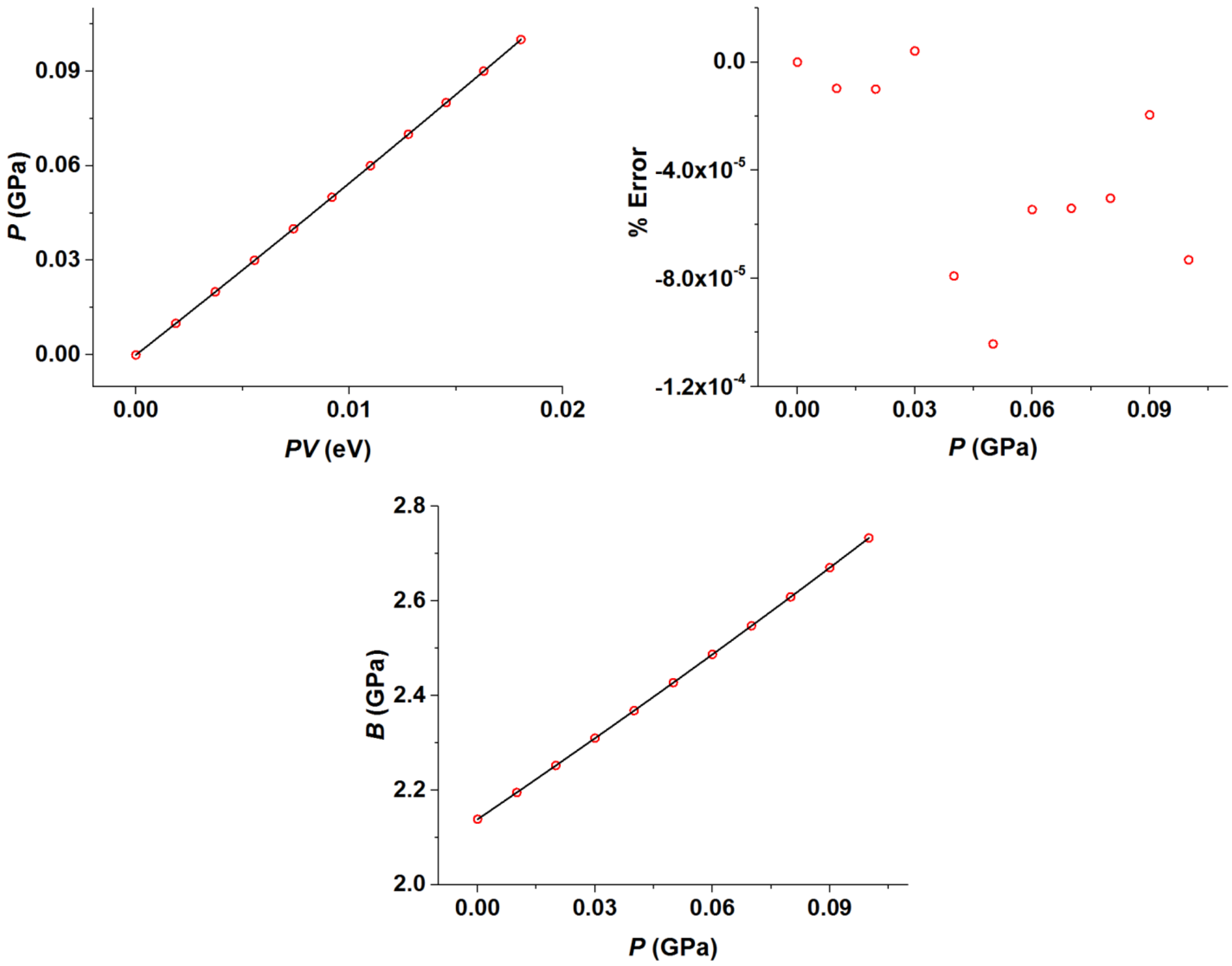

(o) Liquid H_2O at 25 °C

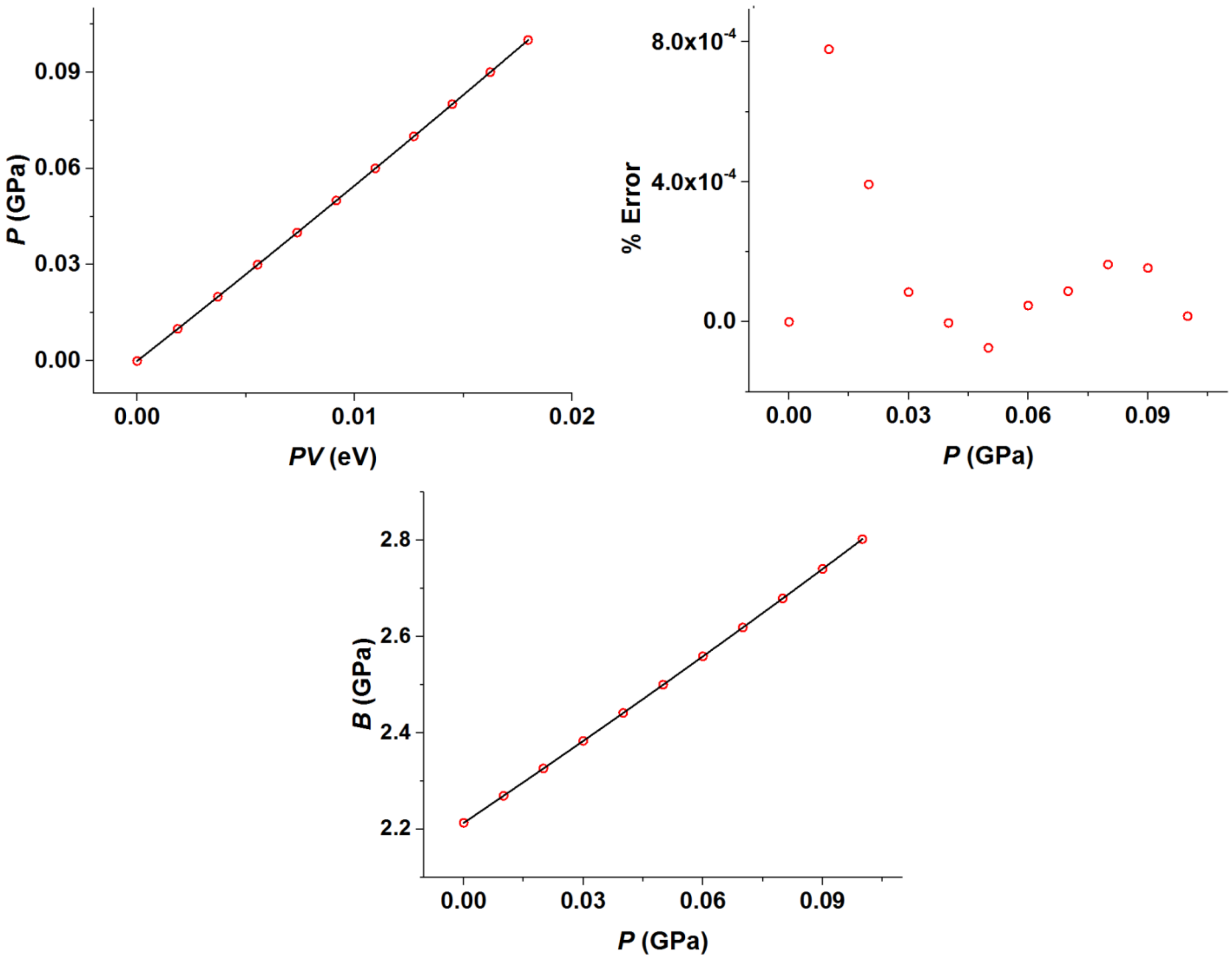

(p) Liquid H_2O at 35 °C

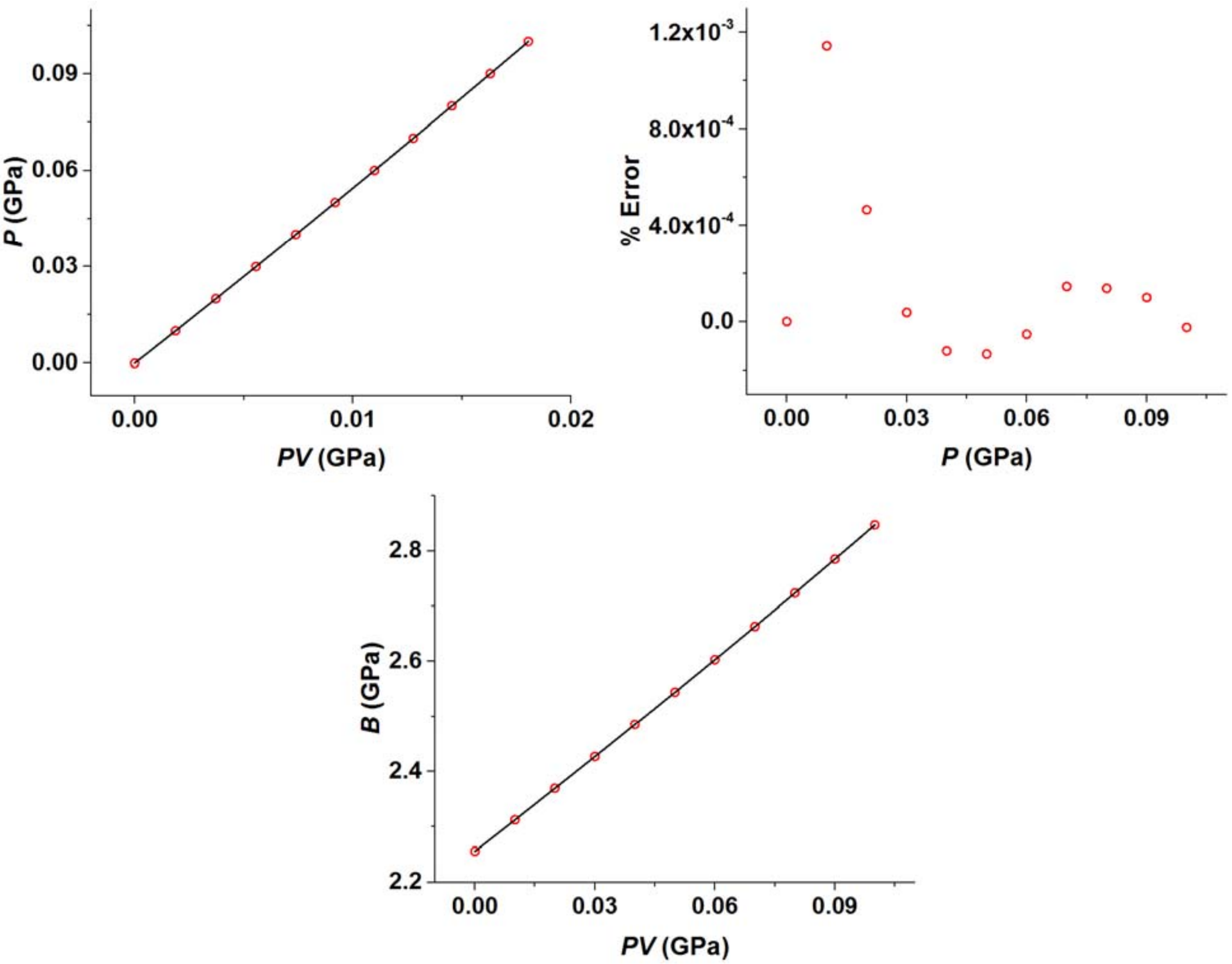

(q) *B*(*P*)-vs-*P* plot for MgO

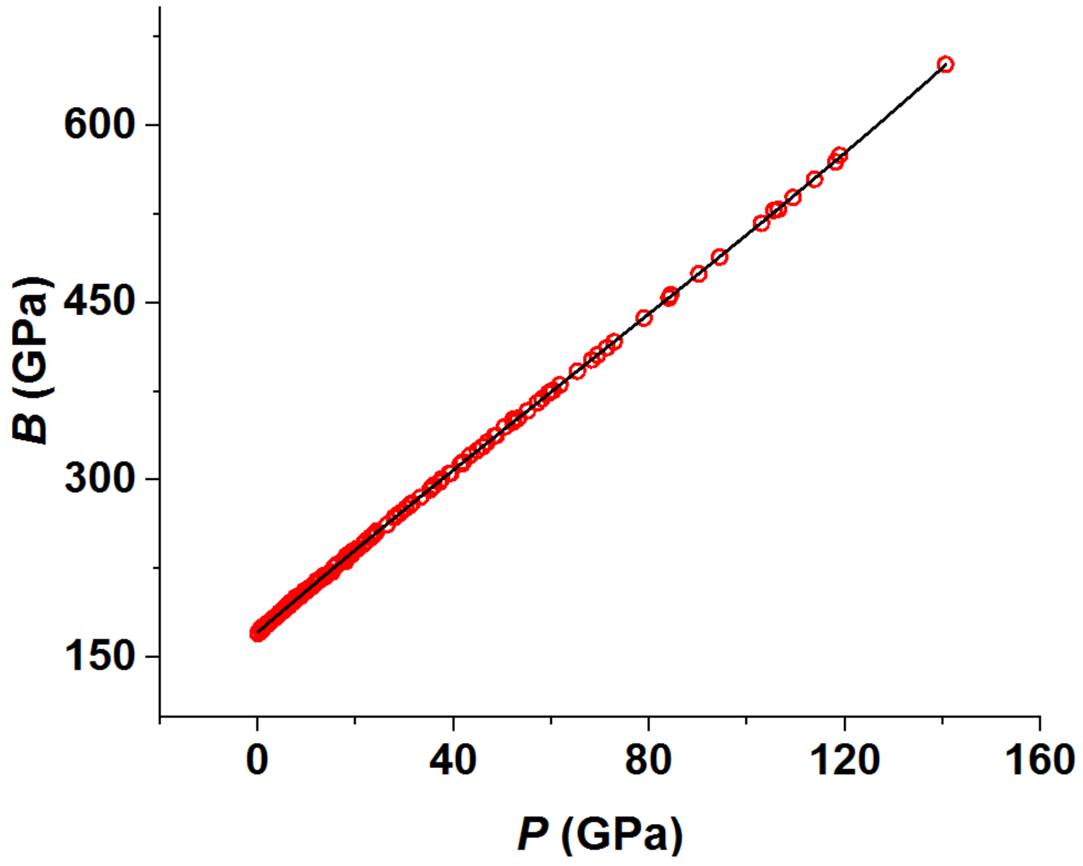

(r) *B*(*P*)-vs-*P* plot for $MgSiO_3$

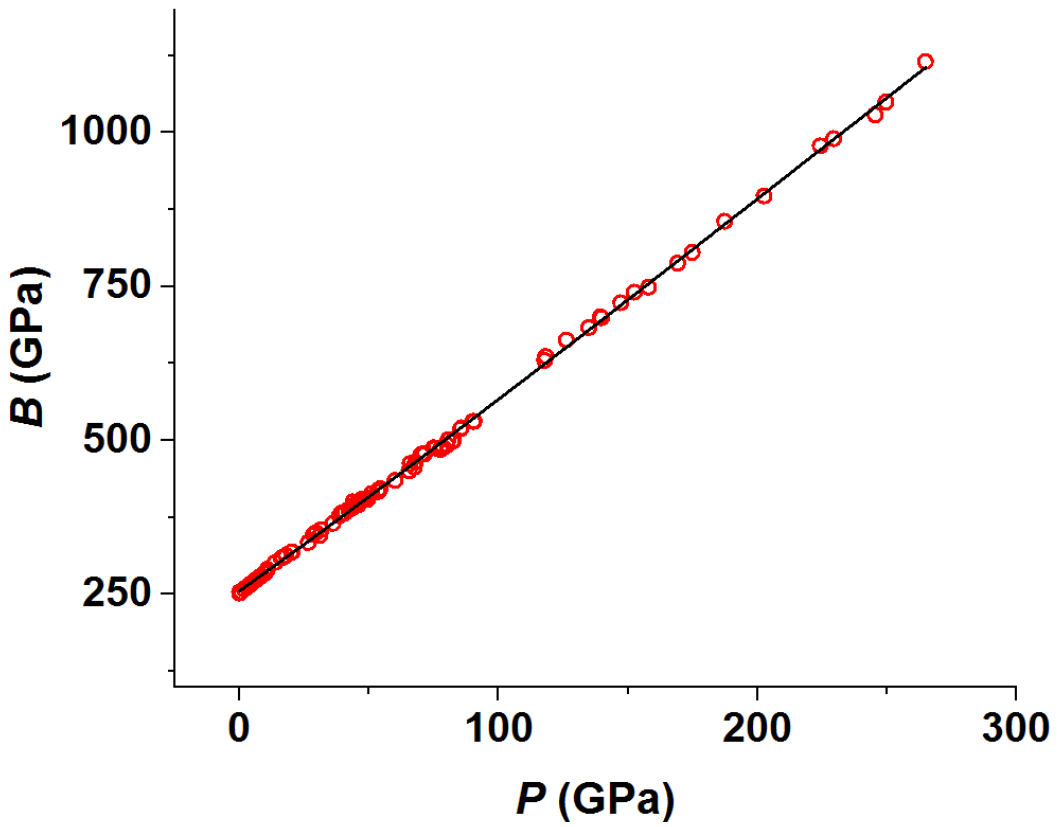

4. Analyses of the $B_{0,expt}$ values reported for the various phases of Te, Se and S

For each phase of chalcogen (Te, Se or S) found in a certain pressure region $P_1 - P_2$, the analysis of the P-vs-V data for the $P_1 - P_2$ region by using the traditional EOS gives rise to the bulk modulus $B_{0,expt}$ and the virtual volume V_0 at $P = 0$ even when P_1 is well above 0. Namely, V_0 is the hypothetical volume the system would have at $P = 0$ if it were to keep the phase found for the $P_1 - P_2$ region down to P = 0. The $B_{0,expt}$ and V_0 values found for various phases of Te, Se and S are listed in Table 1 given below.

Table 1. Simulation of the $B_{0,expt}$ values found for the various phases of Te, Se and S in terms our EOS by using their V_0 values.

	Phase	$B_{0,expt}$ (GPa)	V_0 (Å^3)	$B_{0,calc}$ (GPa)
Te	Phase I	24[14]	31.3[14]	40.6
	Phase II	54[14]	28.0[14]	58.4
	Phas III	57[14]	26.2[14]	67.5
	Phase IV	115[14]	23.6[14]	105.9
	Phase V	425[14]	20.7[14]	604.3
Se	Phase I	48.1[18]	21.6[18]	68.6
	Phase II	63.7[18]	14.5[18]	60.5
	Phase III	263[18]	12.9[18]	249.8
	Phase IV	458[18]	12.1[18]	378.2
S	Orthorhombic	14.5[21]	25.6[21]	20.4
	BCO	21.9[21]	23.9[21]	28.8
	β-Po	30.6[21]	17.2[21]	78.0

In our EQS analysis of the P-vs-V data for a system undergoing several phase transitions, we obtain a single $B(P)$-vs-P relationship (Eq. 3b) valid for the entire pressure region covering

all the phases studied. As already mentioned in the text, Eq. 3b provides only one B_0. In order to simulate the $B_{0,expt}$ values found for the various phases, we proceed as follows:

1) For each phase found in the $P_1 - P_2$ pressure region, we analyze the EOS analysis by using only the P-vs-V data of the $P_1 - P_2$ pressure region. Since the pressure region covering each phase is rather narrow, we employ the quadratic approximation of our EOS, namely, $P = \alpha_1(PV) + \alpha_2(PV)^2$.

2) Using the $P = \alpha_1(PV) + \alpha_2(PV)^2$, we determine the $B(V)$-vs-V relationship using Eq. 3a with $\alpha_3 = 0$.

3) Then we evaluate the $B(V)$ value at $V = V_0$. The resulting $B(V_0)$ is now referred to as the calculated $B_{0,calc}$.

The $B_{0,calc}$ values calculated as described above are listed in Table 1, which exhibits a reasonable agreement with the $B_{0,calc}$ and $B_{0,expt}$ values.
